# Mannose Oligosaccharide‐Conjugated In Situ Pore‐Forming Injectable Hydrogels for Rheumatoid Arthritis Treatment by Reprogramming Macrophage Extracellular Vesicles

**DOI:** 10.1002/smtd.202500605

**Published:** 2025-07-15

**Authors:** Anan Zhang, Yifan Ma, Yutong Liu, Shiyan Dong, Michelle Najarro Torres, Betty Y.S. Kim, Changsheng Liu, Lili Sun, Yuan Yuan, Wen Jiang

**Affiliations:** ^1^ Basic Science Center Project of National Natural Science Foundation of China Frontiers Science Center for Materiobiology and Dynamic Chemistry Key Laboratory for Ultrafine Materials of Ministry of Education and School of Materials Science and Engineering East China University of Science and Technology Shanghai 200237 P. R. China; ^2^ Department of Radiation Oncology MD Anderson Cancer Center Houston TX 77030 USA; ^3^ Department of Neurosurgery The University of Texas MD Anderson Cancer Center Houston TX 77030 USA

**Keywords:** cartilage regeneration, in situ pore‐forming, injectable hydrogels, macrophage extracellular vesicles, rheumatoid arthritis

## Abstract

Rheumatoid arthritis (RA) is a chronic inflammatory disease that causes severe cartilage erosion in joints. Current treatments are limited in accessing a 3D platform that not only supports chondrocyte recovery and new cartilage matrix formation but also effectively modulates the inflammatory environment, particularly through macrophage regulation and extracellular vesicle (EV)‐mediated functions. Here, an injectable hydrogel is developed incorporating mannose oligosaccharide (MOS)‐modified chondroitin sulfate and hyaluronic. This hydrogel forms a porous structure in situ, supporting cell adhesion and matrix production. The MOS groups grafted onto the hydrogel bind to CD206 receptors of macrophages, selectively recruiting and regulating M2 macrophages over an extended period. These macrophages, in turn, release EVs with potent anti‐inflammatory properties, which support new cartilage formation and preservation. This innovative approach addresses a critical gap in RA treatment, offering a novel, cost‐effective, and efficient tissue‐engineering solution with the potential to significantly improve patient outcomes and quality of life.

## Graphical abstract

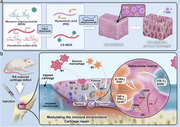

## 1 Introduction

As the global population ages, rheumatoid arthritis (RA)—a common autoimmune disorder—impacts millions by significantly reducing their quality of life.^[^ ^1^ ^]^ RA not only causes joint inflammation but also leads to the progressive breakdown and destruction of joint cartilage.^[^ ^2^ ^]^ The onset and progression of RA are closely linked to the activity of monocytes and macrophages.^[^ ^3^ ^]^ Monocytes infiltrate the joints, where they differentiate into pro‐inflammatory macrophages that secrete excessive pro‐inflammatory cytokines, such as TNF‐α and IL‐1β.^[^ ^4^ ^]^ These cytokines exacerbate inflammation and contribute to cartilage degradation and bone damage. Additionally, macrophage‐secreted metalloproteinases further accelerate cartilage destruction. Current treatments for RA‐related joint damage range from injections of lubricating fluids and analgesics to joint cartilage replacement for more severe cases. However, these interventions do not repair damaged cartilage and primarily aim to alleviate symptoms.^[^ ^5^ ^]^ Due to the lack of effective replacement materials that can mimic natural cartilage, the repair of chondrocytes has become a more desirable alternative in achieving cartilage restoration.

Tissue‐engineered scaffolds possess significant potential by facilitating in situ cartilage repair. Nevertheless, RA presents a challenge due to the uncontrolled inflammatory response that hampers tissue regeneration. As a result, the regulation of the inflammatory environment is critical for the success of cartilage regeneration in RA. Recent advancements in managing inflammation and macrophage activity in RA have demonstrated the potential of macrophage‐secreted extracellular vesicle (EV) therapies to actively modulate the immune environment.^[^ ^6^ ^]^ These therapies function by inducing a phenotypic shift in macrophages from a pro‐inflammatory (M1) state to an anti‐inflammatory (M2) state, thereby modulating the biochemical properties of the secreted EVs that improve inflammation and facilitate tissue repair.^[^ ^7^ ^]^ Further, recent studies highlight the critical role of EVs in mediating communication between macrophages and damaged tissue, including chondrocytes, revealing that EVs may serve as key modulators throughout the repair process.^[^ ^8^ ^]^ Current macrophage‐targeting approaches often rely on expensive or difficult‐to‐manufacture drugs and proteins, such as Disease‐Modifying Antirheumatic Drugs (DMARDs) and cytokines (IL‐21, IL‐35), which not only increase treatment costs but also fail to effectively recruit macrophages to the target site.^[^ ^9^ ^]^ Moreover, conventional systemic drug delivery faces challenges in accurately reaching the target repair site, resulting in drug wastage and potential off‐target effects on other organs. Therefore, there is a pressing need to develop novel, cost‐effective biomaterials that can effectively enhance the quantity and properties of macrophages and their secreted EVs in situ, ensuring targeted and sustained therapeutic effects.

In this study, mannose oligosaccharides (MOS) offer a unique and cost‐effective strategy for reprogramming macrophage EVs. Unlike other macrophage‐targeting approaches, MOS interacts with CD206, a mannose receptor on M2 macrophages, effectively promoting both the recruitment of M2 macrophages and the polarization of M1 macrophages into the M2 phenotype. To the best of our knowledge, such effects of MOS have not been previously explored. This dual functionality of MOS contributes to an elevated secretion of anti‐inflammatory EVs, which play a pivotal role in mediating communication between macrophages and damaged chondrocytes, thereby facilitating cartilage repair.^[^ ^10^ ^]^ Furthermore, the economic advantages of MOS make it a viable option for large‐scale therapeutic applications, addressing a critical limitation of current macrophage‐modulating strategies. However, effectively delivering therapeutic agents without significant loss still remains a challenge. Combining biomaterials with drugs often leads to inefficient delivery, making treatments complex and costly. A simplified and effective delivery strategy for regulating inflammation through the macrophage‐EV pathway is crucial for advancing RA treatment and improving cartilage repair. Therefore, the MOS‐functionalized hydrogel via chemical grafting offers a more effective and sustained therapeutic approach.

In addition to the ability to modulate EV properties, biomaterials need to possess adequate porosity to preserve the morphology and biological functions of chondrocytes.^[^ ^11^ ^]^ Proper porosity is crucial for nutrient exchange, waste removal, and facilitating the infiltration of surrounding cells, all of which are essential for successful tissue regeneration.^[^ ^12^ ^]^ However, incorporating porosity into injectable materials in situ presents significant challenges, particularly in maintaining material consistency and ensuring efficient delivery. In response to these challenges, the development of in situ pore‐forming injectable hydrogels has marked a notable advancement.^[^ ^13^ ^]^ These hydrogels form a porous structure after injection, which adapts to the shape of the damaged area and provides an optimal environment for tissue regeneration. Most importantly, their minimally invasive nature does not compromise effectiveness; rather, they foster a favorable microenvironment for tissue regeneration by providing the necessary space and conditions for cell integration and matrix formation.

**Scheme 1:**
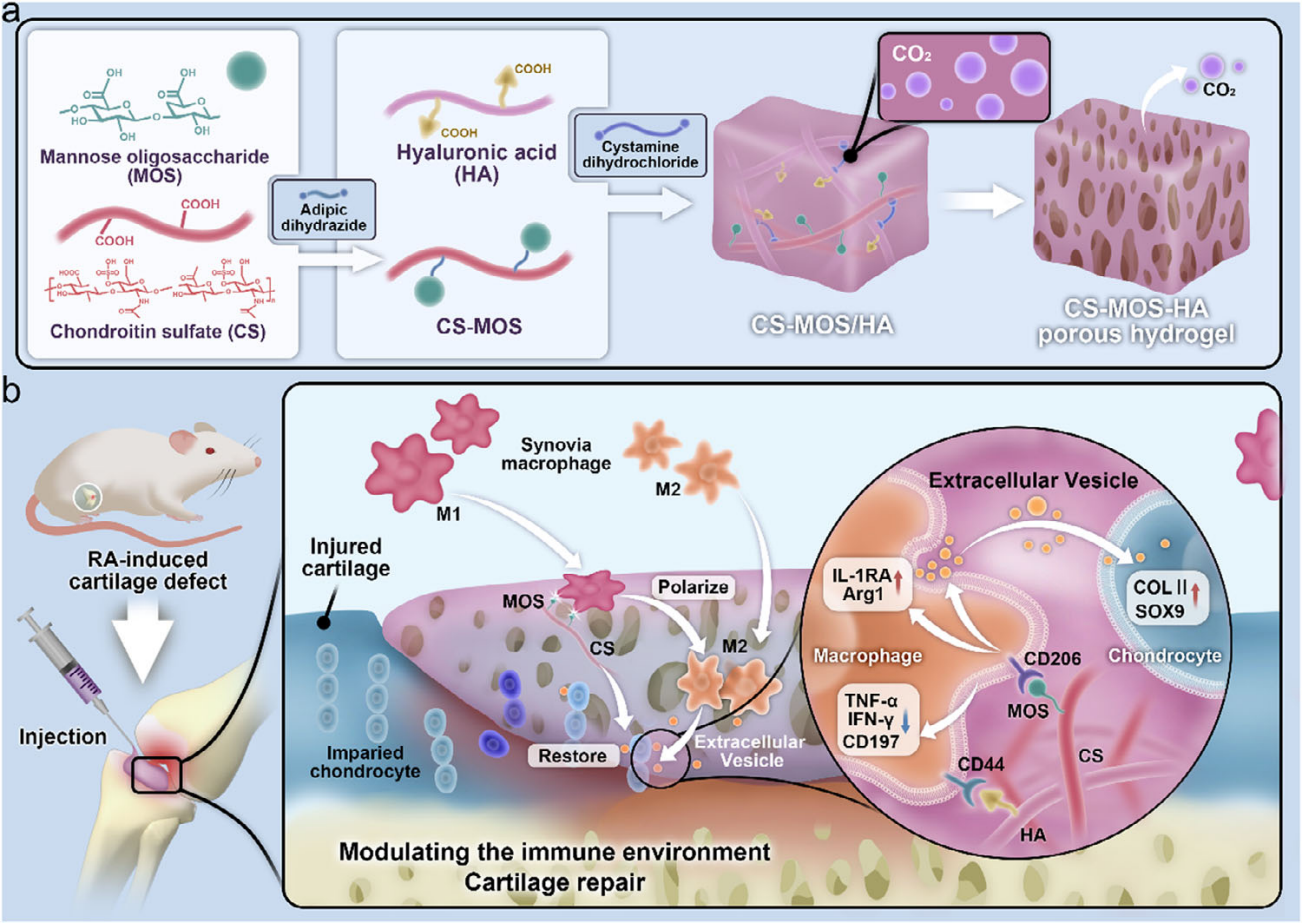
Design of an injectable porous hydrogel modulating macrophages and their secreted EVs for joint defect repair in the pathology of rheumatoid arthritis. a) The solution containing HA and CS‐MOS, upon mixing with a crosslinker solution, undergoes porous hydrogel formation with the escape of generated CO₂ gas. b) Following in situ formation of the hydrogel at the joint defect site in RA rats, the hydrogel effectively recruits macrophages and directs their polarization toward the anti‐inflammatory M2 phenotype. The EVs released by these M2 macrophages then engage with damaged chondrocytes, promoting cartilage regeneration and accelerating defect repair.

Building on these insights, we developed an injectable biomaterial based on naturally occurring cartilage components—hyaluronic acid (HA) and chondroitin sulfate (CS)—for cartilage regeneration in RA. During the hydrogel cross‐linking process, carbon dioxide generated by the reaction between carboxyl groups and diamine crosslinkers facilitated in situ pore formation (**Scheme** **1**a). Moreover, carbon dioxide release has been demonstrated to attenuate inflammation through the NF‐κB signaling pathway.^[^ ^14^ ^]^ Therefore, hydrogels with in situ CO₂ release properties are well‐suited for RA treatment. To further enhance the therapeutic potential, MOS groups were grafted on polymer chains of the hydrogel, enabling macrophage phenotype modulation and regulation of their released EVs, thereby enhancing the immunomodulatory and reparative properties of our biomaterials (Scheme 1b). This dual strategy provides structural integrity for cartilage repair while also promoting a favorable immune environment for healing. In vitro experiments were conducted to isolate EVs from macrophages and evaluate their effects on RA chondrocytes. We then assessed the hydrogel's immunomodulatory effects and its ability to repair cartilage in a rat model of collagen‐induced arthritis (CIA), comparing the results with and without EV inhibitors.

## 2 Results and Discussion

### 2.1 MOS‐Conjugated In Situ Pore‐Forming Injectable Hydrogel Preparation

In this study, hyaluronic acid and chondroitin sulfate, both rich in carboxyl groups and pivotal components of cartilage, were selected as the primary materials. To enhance the immunomodulatory properties, MOS groups were first grafted onto a portion of the carboxyl groups of chondroitin sulfate. Subsequently, the carboxyl groups of both hyaluronic acid and chondroitin sulfate were conjugated with the amino groups of cystamine dihydrochloride via a reaction catalyzed by 1‐ethyl‐3‐(3‐dimethylaminopropyl) carbodiimide (EDC) and N‐hydroxysuccinimide (NHS), leading to the formation of a hydrogel. During the activation of the carboxyl groups by EDC and NHS, carbon dioxide was released as a byproduct. This gas‐generation process enabled the pre‐crosslinked injectable mixture to form pores in situ within the body as the cross‐linking progressed.

**Figure 1:**
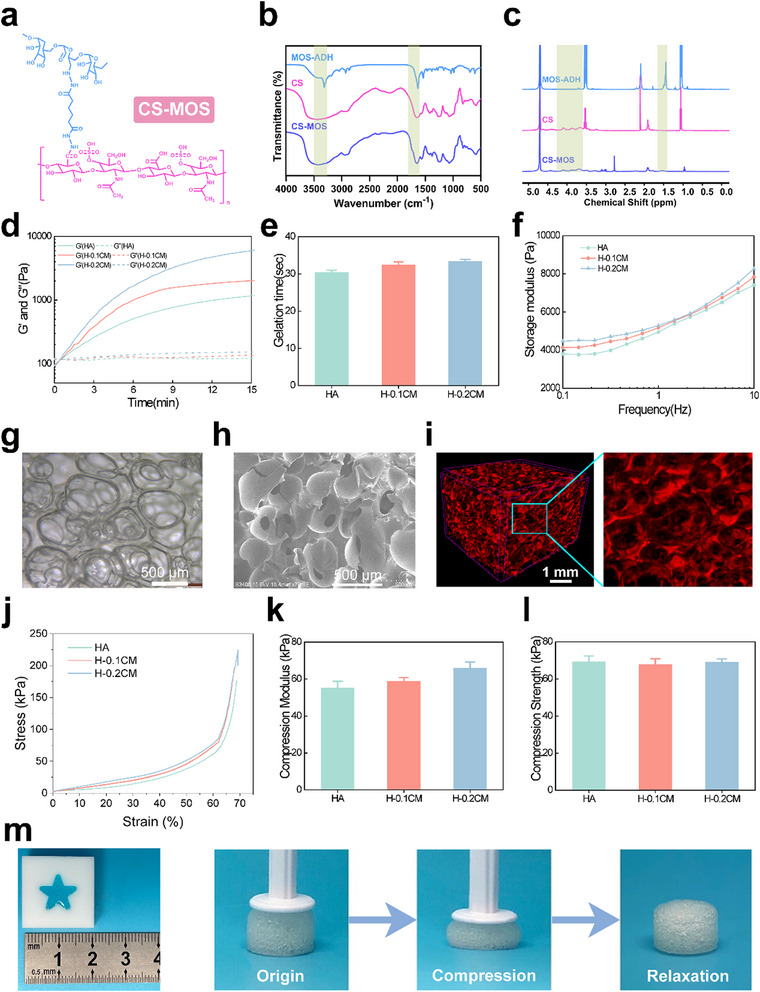
Characterization of hydrogels. a) Schematic representation of the chemical structure of CS‐MOS. b) FTIR spectra of MOS‐ADH, CS, and CS‐MOS. c) ^1^H NMR spectra of MOS‐ADH, CS, and CS‐MOS. d) Storage modulus (G′) and loss modulus (G″) of hydrogels with varying MOS content as a function of time (37 °C, 1 rad·s^−1^) (*n* = 3). e) Gelation time of different hydrogels (*n* = 3). f) Storage modulus (G′) of different hydrogels in terms of frequency (37 °C, 1% strain) (*n* = 3). g–i) Representative pore structure of hydrogels observed by inverted microscopy (g), cryo‐SEM (h), and micro‐CT (i). j–l) Compression tests of the different hydrogels. Stress–strain curve (j), compression modulus (k), and compression strength (l) of the hydrogels (*n* = 3). m) Hydrogel appearance under excessive compression. All data are presented as mean ± SD.

To prepare MOS‐conjugated chondroitin sulfate (CS‐MOS), the glycosidic units of MOS were first oxidized with sodium periodate (NaIO₄) to produce oxidized MOS (OxMOS), which contains aldehyde groups. These aldehyde groups then reacted with the amino groups of adipic dihydrazide (ADH) to form Schiff bases. Sodium borohydride (NaBH₃CN) was subsequently employed as a reducing agent to stabilize the imine bonds in the Schiff bases, converting them into stable amine linkages and thereby introducing amine groups into OxMOS, resulting in ADH‐conjugated MOS (MOS‐ADH). The remaining hydrazine groups on ADH were then used to react with the carboxyl groups of CS, leading to the synthesis of CS‐MOS (**Figure** **1**a).

Each product was characterized using infrared (IR) spectroscopy and nuclear magnetic resonance (NMR). The ¹H NMR spectrum of MOS‐ADH (Figure 1c) displays distinct peaks corresponding to various functional groups: 1.0–1.1 ppm (MOS), 1.4–1.6 ppm (ADH), 2.0–2.2 ppm (ADH), 3.47–3.57 ppm (MOS), and 4.70 ppm (D₂O). The presence of peaks from both MOS and ADH confirms the successful grafting of ADH onto MOS. Similarly, the FTIR and ¹H NMR spectra of CS‐MOS also show characteristic peaks from both CS and MOS (Figure 1b,c). The FTIR spectrum of CS‐MOS exhibited a vibrational peak for the N‐H bond of the hydrazide group in ADH at 3300–3500 cm⁻¹, as well as the asymmetric stretching vibration of the COO‐ group in CS from MOS at 1700–1800 cm⁻¹. The ¹H NMR spectrum of CS‐MOS shows the methylene (‐CH₂‐) peaks of ADH at 1.4–1.6 ppm and 2.0–2.2 ppm. These findings confirm the successful grafting of MOS onto CS via the ADH linker, validating the successful synthesis and functional integration of these biopolymers. The entire CS‐MOS synthesis process is illustrated in Figure S1 (Supporting Information).

Finally, the synthesized CS‐MOS was crosslinked with HA using EDC and NHS, resulting in the hydrogel formation. Both HA and CS‐MOS contain carboxyl groups capable of reacting with the primary amine groups at both ends of cystamine dihydrochloride, thus initiating the crosslinking process. EDC and NHS activate the carboxyl groups, and during this activation, CO₂ gas is produced as bubbles. These gas bubbles are trapped within the hydrogel matrix due to the viscosity of the pre‐gel solution and the rapid gelation process, thereby creating a porous structure. Our method successfully results in injectable, porous hydrogels. By adjusting the amount of MOS incorporated, two hydrogel formulations (H‐0.1CM and H‐0.2CM) were prepared.

### 2.2 The Preparation and Characterization of In Situ Pore‐Forming Injectable H/CS‐MOS Hydrogels

Building on the successful preparation of the materials, the injectability characteristics of the H/CS‐MOS hydrogel were evaluated by measuring gelation time. The gelation time was determined from the rheological time sweep curves, which marked the point where the storage modulus (G′) and loss modulus (G″) intersected. As shown in Figure 1d, at 37 °C the G′ of the hydrogel had a low starting point but increased rapidly, reaching the crossover point with G″ within a short period. After 15 min G′ stabilizes, indicating a rapid gelation process. Before the intersection point in the figure, G′ remains lower than G″, indicating that the pre‐gel solution retains its fluidity, ensuring excellent injectability. As crosslinking progresses, G′ increases, signifying the formation of a stable cross‐linked network. The gelation times for H, H‐0.1CM, and H‐0.2CM hydrogels were all ≈30 s (Figure 1e), allowing for direct injection into tissue defects due to their short gelation times.

The porosity of the hydrogels was assessed by examining the microstructural morphology of lyophilized H/CS‐MOS hydrogels using scanning electron microscopy (SEM). As illustrated in Figure S2 (Supporting Information), all three hydrogel groups exhibited interconnected macroporous structures. To further investigate the in situ formation of these porous structures, non‐lyophilized H/CS‐MOS hydrogels were analyzed using both an inverted microscope and cryo‐SEM (Figure 1g,h; Figure S3, Supporting Information). Both imaging techniques revealed similar interconnected macroporous structures, confirming that the hydrogels retained their porous integrity after lyophilization. These findings suggest that the hydrogels form interconnected “transportation highways” through in situ foaming, which could facilitate cell infiltration and growth following injection.

To further quantify the pore structure, Micro‐CT scanning was performed on the H/CS‐MOS hydrogels (Figure 1i). The results indicated that the HA group, H‐0.1CM group, and H‐0.2CM group had similar pore sizes of 491 ± 25 µm, 487 ± 36 µm, and 484 ± 54 µm, respectively, with a total porosity of ≈85% (Table S1, Supporting Information). These findings confirm that the H/CS‐MOS hydrogels possess a highly porous, interconnected structure that supports chondrocyte migration, adhesion, and the maintenance of their spheroidal shape. Moreover, the micrometer‐sized pores and high porosity of these hydrogels make them ideal candidates for cartilage tissue repair applications.

In the advanced stages of rheumatoid arthritis, significant and deep defects can develop in both cartilage and bone at the joints, compromising the surrounding mechanical structure. Simple injections of medicated fluids or microspheres are insufficient to restore mechanical support. Therefore, biomaterials are required to ensure structural integrity and mechanical stability at the defect sites. Compression stress–strain tests were performed to assess the compressive behavior of the three hydrogel groups under identical conditions. The results revealed no significant differences among the groups, with all hydrogels being compressible to ≈70% strain (Figure 1j; Figure S4, Supporting Information). As shown in Figure 1m, the hydrogels exhibited excellent mechanical stability and recovery from deformation. When subjected to excessive compression at higher strain rates, the hydrogels fully returned to their original state, indicating strong compression resistance. This resilience suggests that the hydrogels possess robust compressive properties, likely attributed to their porous structure. The interconnected macroporous architecture and high porosity enable the hydrogels to withstand higher deformation rates while maintaining their integrity and recovering from compressive forces. To evaluate H/CS‐MOS hydrogel response to increasing loads, a rheological frequency sweep experiment was conducted. The steady increase in the storage modulus (G′) with increasing frequency (Figure 1f) indicates the material's viscoelastic properties, which are known to support the maintenance of chondrocyte differentiation.^[^ ^15^ ^]^

These characterization results collectively demonstrate that the prepared hydrogels exhibit excellent injectability, as well as the formation of a well‐defined porous structure upon injection and crosslinking. Furthermore, the hydrogels possess mechanical properties that support chondrocyte viability, resist external forces, and maintain structural integrity at defect sites. The various key features of the H/CS‐MOS hydrogels make them a highly promising injectable biomaterial for cartilage tissue repair in the treatment of RA.

### 2.3 H/CS‐MOS Hydrogels Enhanced Recruitment of M2 Macrophages

Macrophages, a key class of immune cells, are essential for maintaining tissue homeostasis and overall health. Studies have shown that M2‐type macrophages can modulate the microenvironment at injury sites, promoting tissue repair and regeneration.^[^ ^16^ ^]^ The HA component of the H/CS‐MOS hydrogel contains recognition sites for the CD44 receptor on cell surfaces, facilitating cell adhesion and regulating cellular behavior.^[^ ^17^ ^]^ MOS binds to the CD206 protein on macrophage surfaces, enhancing their adhesion and activating CD206 expression, thereby influencing macrophage activity.

An in vivo subcutaneous implantation model using C57BL/6 mice was employed to investigate the regulation of macrophage adhesion by the H/CS‐MOS hydrogel. The materials were retrieved from the mice four days post‐implantation. Adherent cells on the material surfaces were characterized using DAPI staining, and the recruited macrophages were identified through F4/80 immunolabeling. Figure S5a (Supporting Information) shows that macrophage cells mainly adhered to the hydrogel surfaces four days after implantation. The H‐0.1CM and H‐0.2CM groups exhibited stronger F4/80 fluorescence compared to the HA group, indicating a greater number of macrophages adhered to the H/CS‐MOS hydrogel surfaces. The H‐0.2CM group showed the highest fluorescence intensity, suggesting that a higher CS‐MOS content further enhances macrophage recruitment. Immunofluorescence staining was also performed to assess CD44 and CD206 protein expression on cells adhering to the surfaces of the H/CS‐MOS hydrogel. As shown in Figure S5b (Supporting Information), the H‐0.1CM and H‐0.2CM groups, which incorporated CS‐MOS, displayed significantly higher fluorescence expression of CD206 and CD44 compared to the pure HA hydrogel group, with expression increasing as the CS‐MOS content increased. Therefore, the H‐0.2CM group was selected as the primary experimental group for subsequent studies and is referred to as the H/CS‐MOS group.

**Figure 2:**
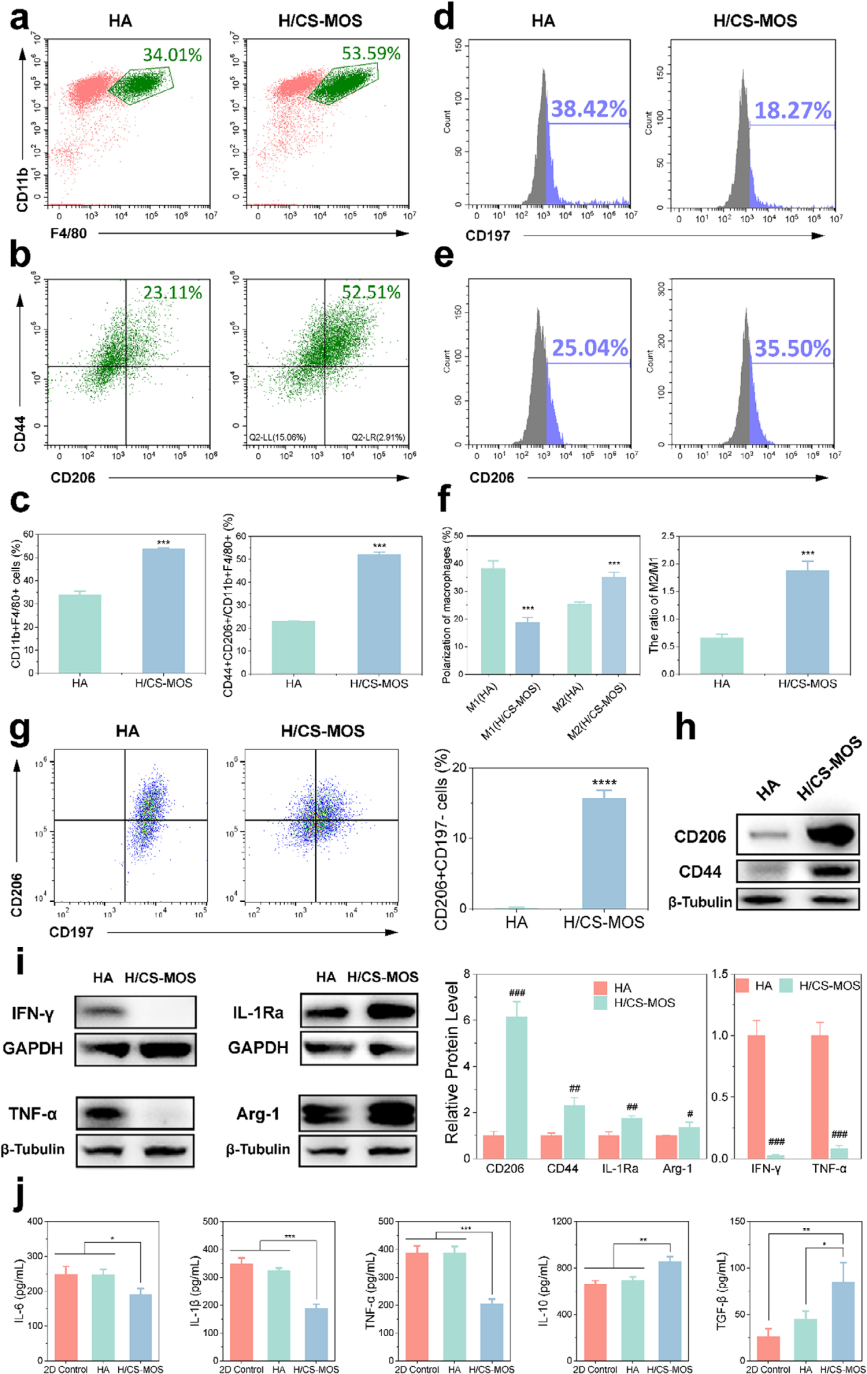
H/CS‐MOS hydrogels regulate the recruitment and polarization of macrophages. a–c) Flow cytometric analysis showing the proportion of the macrophages among the recruited cells (a) and the proportion of the M2 macrophages among all the macrophages (b) along with their quantitative comparison (c) (*n* = 3). d–f) Flow cytometry analysis of the polarization of the macrophages, indicated by CD197 (M1 phenotype, d) and CD206 (M2 phenotype, e), along with their quantitative comparison (f) (*n* = 3). g) Flow cytometry analysis showing the polarization shift of M1 macrophages (induced with LPS and IFN‐γ) cultured on the different scaffolds, along with their quantitative comparison (*n* = 3). h,i) Western blot analysis of protein levels of CD206, CD44, pro‐inflammatory factors (IFN‐γ and TNF‐α), anti‐inflammatory factor (IL‐1Ra), and Arg‐1, with quantification normalized to the HA group (*n* = 3). j) Quantitative analysis of inflammatory cytokine levels of IL‐6, IL‐1β, TNF‐α, IL‐10 and TGF‐β (*n* = 3). All data are presented as mean ± SD. (The *p* values were determined using unpaired two‐tailed t tests for two‐group comparisons and one‐way ANOVA followed by Tukey's post hoc test for multiple‐group comparisons. ^*^ *p* < 0.05, ^**^ *p* < 0.01, ^***^ *p* < 0.001, ^****^ *p* < 0.0001, ^#^ *p* < 0.05, ^##^ *p* < 0.01, ^###^ *p* < 0.001, ^#^ indicates a comparison with the HA group within the same protein experimental group.

Flow cytometry was used for further analysis of cells recruited by the H/CS‐MOS hydrogel in vivo. As shown in **Figure** **2**a, the number of macrophages recruited to the hydrogel surface significantly increased in the H/CS‐MOS group, rising from 34.01% in the HA group to 53.59% in the H/CS‐MOS group. Among these recruited macrophages, the percentage expressing both CD44 and CD206 increased from 23.11% in the HA group to 52.21% in the H/CS‐MOS group (Figure 2b). Specifically, the total count of macrophages (CD11b+F4/80+) in the H/CS‐MOS group was double in comparison to that observed in the HA group, and the total count of macrophages expressing both CD44 and CD206 (CD44+CD206+CD11b+F4/80+) was roughly four times higher (Figure 2c). These findings indicate a marked increase in both macrophage recruitment and CD44/CD206 expression in the H/CS‐MOS group.

Since CD206 is a known marker of M2 macrophage polarization, macrophage M1 and M2 phenotype polarization was assessed using flow cytometry. This analysis assessed the expression of CD197 as the M1 marker and CD206 as the M2 marker, on cells adhering to the material's surface. The results shown in Figure 2d,e indicate that the H/CS‐MOS hydrogel significantly decreased CD197 expression from 38.24% to 18.27% compared to the HA group, while simultaneously increasing CD206 expression. The M2/M1 polarization ratio was calculated for each group. The HA group had an M2/M1 ratio of ≈0.6, indicating a predominance of the M1 phenotype over the M2 phenotype. In contrast, the H/CS‐MOS group had an M2/M1 ratio nearing 2, reflecting a predominant presence of M2 phenotype macrophages (Figure 2f).

### 2.4 H/CS‐MOS Hydrogel Induced Macrophage Transition Toward M2 Phenotype by Regulating Macrophage Metabolism

In the inflammatory environment of RA, a high number of macrophages accumulate around the defect, typically adopting a pro‐inflammatory M1 phenotype.^[^ ^18^ ^]^ This M1 phenotype exacerbates cartilage deterioration in the inflamed environment; therefore, a shift from M1 to M2 is indispensable to lower inflammation.^[^ ^19^ ^]^ M1 polarization was induced in bone marrow‐derived macrophage (BMM) cells by incubation with LPS and IFN‐γ, followed by culture within the hydrogel. Flow cytometry was used to assess whether the H/CS‐MOS hydrogel could influence macrophage polarization under inflammatory conditions. As shown in Figure 2g, the HA group exhibited minimal change in the M1 macrophage phenotype, whereas the percentage of M2 macrophages (CD206+CD197‐ cells) significantly increased to 15.67 ± 1.15% in the H/CS‐MOS group. These results suggest that incorporating CS‐MOS into the hydrogels not only attracts M2 macrophages but also promotes the conversion of M1 to M2 macrophages in inflammatory environments. Further assessment of macrophage levels of pro‐ and anti‐inflammatory proteins was performed using Western blotting (Figure 2h,i). The H/CS‐MOS hydrogel significantly reduced pro‐inflammatory IFN‐γ and TNF‐α levels compared to the HA group, while increasing the levels of the M2 marker CD206, as well as the anti‐inflammatory proteins Arg1 and IL‐1Ra. To further validate the regulatory effect of H/CS‐MOS on the inflammatory microenvironment, ELISA tests were conducted to measure cytokine levels in the cell culture supernatant after co‐culture with hydrogels. As shown in Figure 2j,k, the H/CS‐MOS group significantly decreased pro‐inflammatory cytokines IL‐6, IL‐1β, and TNF‐α, while markedly upregulating anti‐inflammatory cytokines IL‐10 and TGF‐β, compared to the hydrogel‐free and HA groups. These findings collectively highlight the ability of the H/CS‐MOS hydrogel to modulate macrophage polarization and reduce inflammation associated with RA.

The stimulation of the H/CS‐MOS hydrogel altered macrophage phenotypes, shifting them from the M1 to the M2 state, and the underlying mechanisms warrant further investigation. While some studies have shown that targeting the mannose receptor can induce phenotypic changes in immune cells, the specific mechanisms remain poorly understood. It has been reported that the activation of pattern recognition receptors (PRRs) in immune cells influences cellular metabolic processes.^[^ ^20^ ^]^ Additionally, immunometabolism plays a central role in regulating macrophage activation states and associated functions.^[^ ^21^ ^]^ Compared to M1 polarization, M2 polarization is associated with metabolic pathways that generate higher energy production. M2 macrophages primarily produce energy through oxidative phosphorylation (OXPHOS), while M1 macrophages rely mainly on glycolysis.^[^ ^22^ ^]^ Therefore, the MR‐targeting approach may regulate M2 polarization by influencing macrophage energy metabolism.

**Figure 3:**
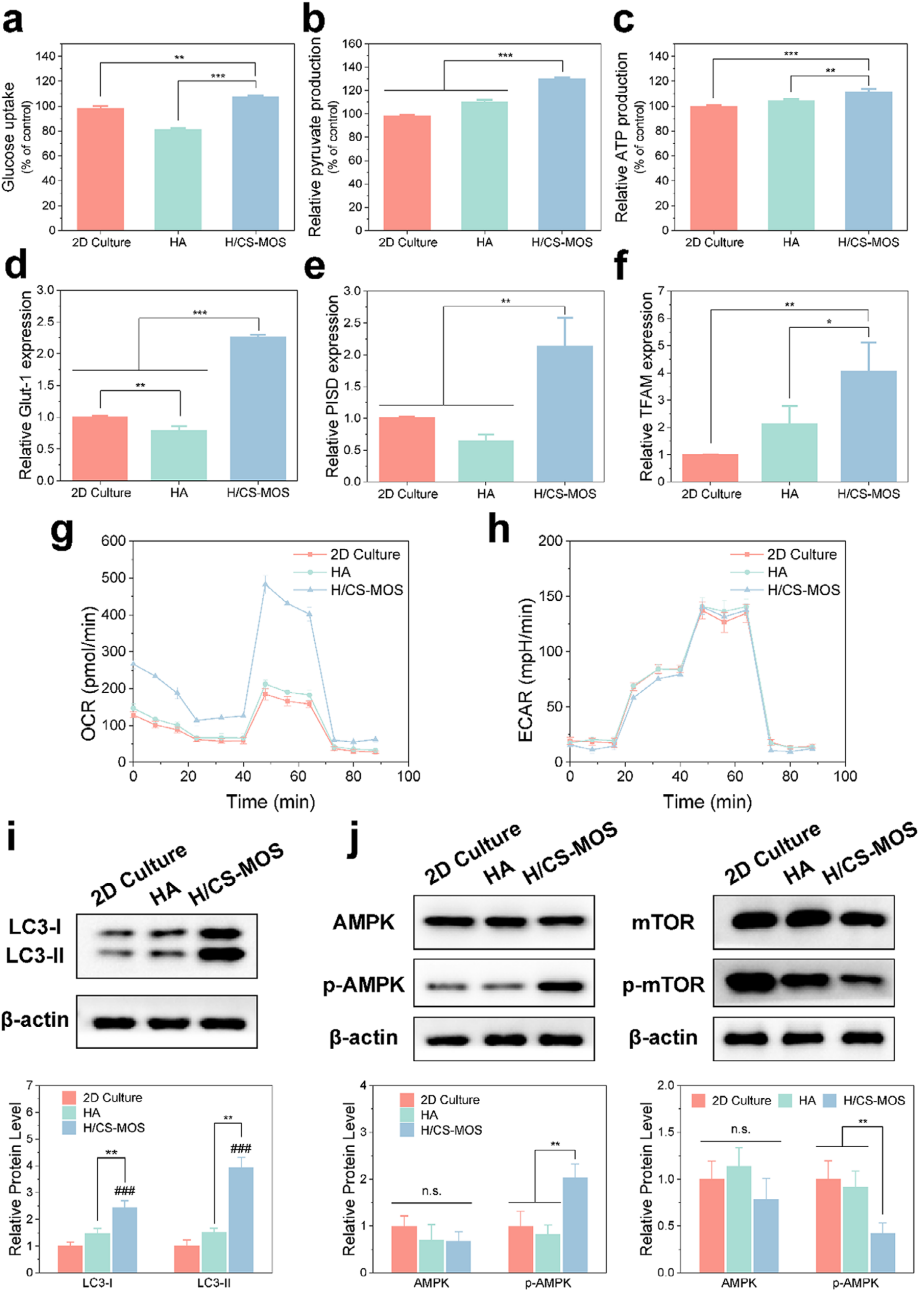
H/CS‐MOS hydrogels induce macrophage M2 polarization by regulating cell metabolism. a–c) Glucose uptake (a), pyruvate acid level (b), and ATP level (c) of macrophages (2D culture as the control group) (*n* = 3). d,e) mRNA levels of glucose uptake‐related (Glut‐1), and OXPHOS‐related (PISD and TFAM), shown by RT‐PCR, with data normalized to the 2D Culture group (*n* = 3). g,h) OCR and ECAR analysis of macrophages (*n* = 3). i,j) Western blot analysis of LC3, AMPK, p‐AMPK, mTOR, and p‐mTOR protein levels, with quantification normalized to the 2D Culture group (*n* = 3). All data are presented as mean ± SD (The *p* values were determined using two‐tailed one‐way ANOVA with a Tukey post hoc test. ^*^ *p* < 0.05, ^**^ *p* < 0.01, ^***^ *p* < 0.001, ^###^ *p* < 0.001, ^#^ indicates a comparison with the 2D Culture group within the same protein experimental group, “n.s.”: no significant difference).

M1 macrophages served as the control group to evaluate the effects of hydrogel co‐culture on macrophage glucose metabolism. As shown in **Figure** **3**a–c, macrophages cultured with H/CS‐MOS exhibited enhanced glucose uptake, along with increased production of pyruvate and adenosine triphosphate (ATP), compared to the other two groups. These results suggest that H/CS‐MOS induces increased energy demands and metabolic activity in macrophages.

Furthermore, the primary pathways of macrophage glucose metabolism were characterized. The RT‐PCR results shown in Figure 3d–f revealed upregulation of genes related to glucose uptake (Glut‐1) and OXPHOS (PISD and TFAM) in the H/CS‐MOS group, indicating increased energy mobilization and OXPHOS activity in H/CS‐MOS‐treated macrophages. Mitochondrial stress tests and glycolysis rate assays were further conducted to assess mitochondrial function and glycolysis, respectively, by measuring oxygen consumption rate (OCR) and extracellular acidification rate (ECAR). Figure 3g,h showed the enhanced mitochondrial function for H/CS‐MOS group, indicating energy production through aerobic metabolism, while glycolysis levels remained nearly unchanged.

Beyond the enhancement of energy metabolism, the role of autophagy in regulating macrophage function was further explored. Microtubule‐associated protein light chain 3 (LC3) is a key autophagy marker. As shown in Figure 3i, Western blot results demonstrated that the H/CS‐MOS hydrogel significantly increased the expression of LC3‐II and the LC3‐II/LC3‐I ratio (Figure S6, Supporting Information) in macrophages, compared to the other two groups, indicating enhanced autophagy levels.

Finally, the expression of AMPK and mTOR proteins in hydrogel‐cultured macrophages was examined. AMPK regulates cellular energy balance by modulating metabolic pathways such as glucose metabolism and autophagy, while downregulating mTOR to activate autophagy. The western blot results in Figure 3j revealed that the H/CS‐MOS group exhibited significantly increased levels of phosphorylated AMPK (p‐AMPK) and decreased levels of phosphorylated mTOR (p‐mTOR) compared to the other groups.

### 2.5 H/CS‐MOS Hydrogel Reprogrammed Macrophage‐Secreted EVs for Promoting Biosynthesis of RA Chondrocytes

Immune cells lack the ability to differentiate into chondrocytes and, therefore, are unable to contribute to the repair of tissue directly.^[^ ^23^ ^]^ As a result, in the inflamed environment of RA, EVs are indispensable for cell signaling and communication.^[^ ^6^, ^8^ ^]^ In particular, EVs secreted by macrophages on chondrocytes are crucial for enhancing cartilage repair.

**Figure 4:**
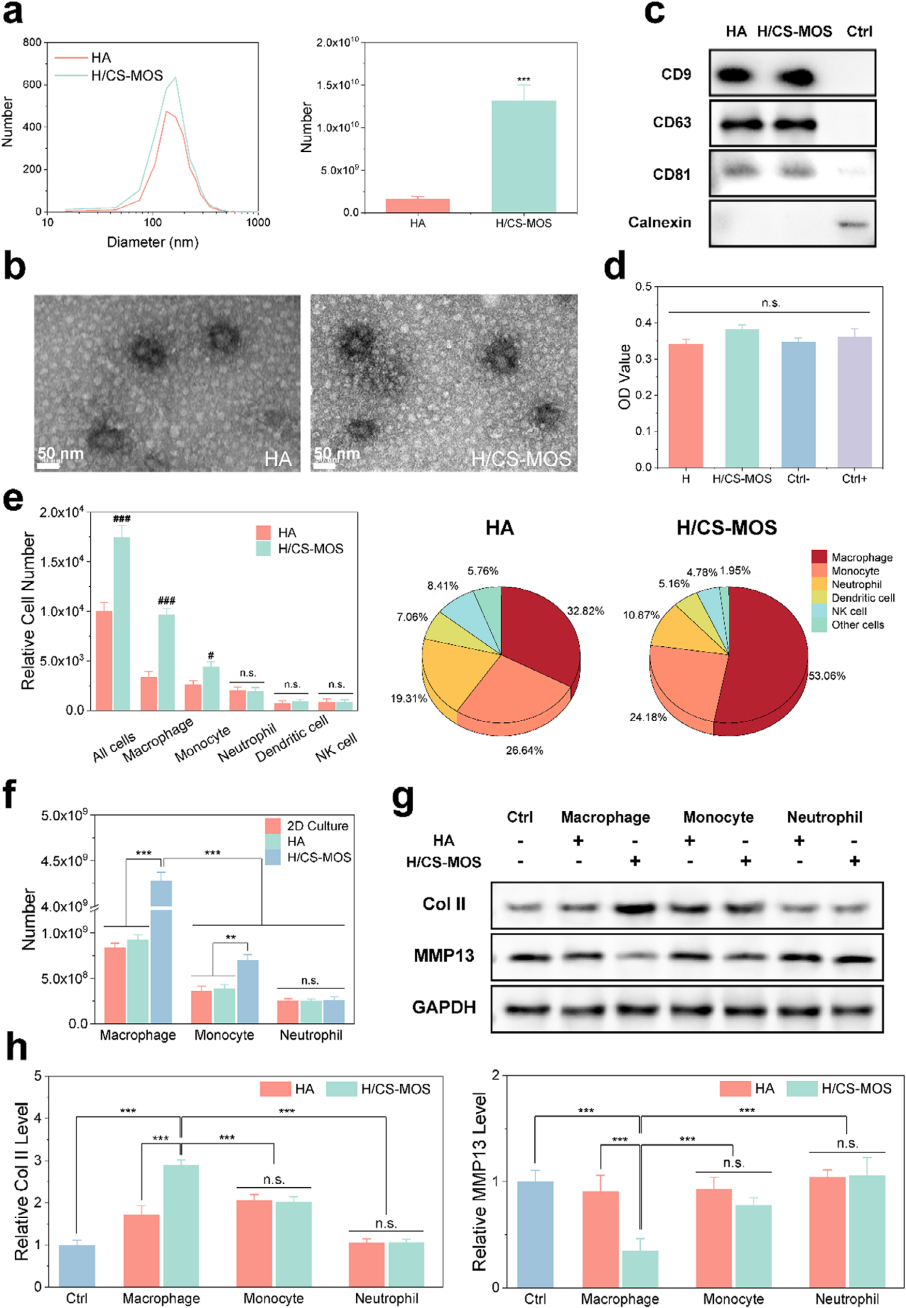
H/CS‐MOS hydrogel promotes macrophage EV production by enhancing macrophage recruitment and secretion capacity. a) Particle size distribution and relative quantity of EVs secreted by cells recruited on the scaffolds measured using NTA (*n* = 3). b) Representative TEM images of the EVs. c) Expression of EV surfaces marker proteins CD9, CD63, CD81, and the absence of the non‐EV protein Calnexin. d) Effects of EVs of H group and H/CS‐MOS group on RA chondrocytes viability (*n* = 3). e) Relative quantity and proportion of various recruited cells in vivo for HA and H/CS‐MOS hydrogels analyzed by flow cytometry (*n* = 3). f) Relative EV secretion from macrophages, monocytes, and neutrophils after co‐culture with HA and H/CS‐MOS hydrogels measured using NTA (*n* = 3). g) Expression levels of Col II and MMP13 in RA chondrocytes after co‐culture with EVs derived from different cells co‐cultured with HA and H/CS‐MOS hydrogels. h) Relative protein expression based on Western blot analysis, with data normalized to the control group (*n* = 3). All data are presented as mean ± SD. (The *p* values were determined using unpaired two‐tailed t tests for two‐group comparisons and one‐way ANOVA followed by Tukey's post hoc test for multiple‐group comparisons. ^**^ *p* < 0.01, ^***^ *p* < 0.001, ^#^ *p* < 0.05, ^###^ *p* < 0.001, # indicates a comparison with the HA group within the same cell experimental group, “n.s.”: no significant difference).

Both in vivo and in vitro studies demonstrate that the H/CS‐MOS hydrogel effectively recruits a significant number of macrophages and promotes their polarization from the M1 to the M2 phenotype. Subsequently, EVs secreted by cells adhering to the surface of the H/CS‐MOS hydrogels were harvested and analyzed four days after injection in vivo. Nanoparticle tracking analysis (**Figure** **4**a) measured the size of these EVs, revealing an average diameter of ≈150 nm following ultracentrifugation. Statistical analysis showed that the quantity of EVs secreted in the H/CS‐MOS group was ≈8.25 times greater than in the HA group. The TEM images (Figure 4b) confirmed the EVs' spherical shape. The presence of EV markers CD9, CD63, and CD81, as well as the non‐EV protein Calnexin, was determined by Western blotting.^[^ ^24^ ^]^ As shown in Figure 4c and Figure S7 (Supporting Information), EVs from both the HA and H/CS‐MOS groups were positive for CD9, CD63, and CD81, while Calnexin was only detected in BMM cells.

EVs can influence the behavior of recipient cells through the RNA they contain.^[^ ^6^, ^8^, ^25^ ^]^ To assess their potential for cartilage repair, their effects on cell proliferation were investigated. After RA chondrocytes were co‐cultured with EVs from the HA and H/CS‐MOS groups on hydrogels for 3 days, the impact on proliferation was evaluated using CCK‐8 assays. As shown in Figure 4d, chondrocyte proliferation was significantly promoted by the EVs secreted from cells on the H/CS‐MOS hydrogel, indicating strong biological activity that facilitates chondrocyte repair.

The mechanisms underlying the enhanced EV secretion and its relationship with macrophages were further investigated. Since the cellular origin of each EV could not be directly determined, flow cytometric analysis was performed on the cells recruited and adhered to the hydrogels. Several representative immune cell types, including macrophages, monocytes, neutrophils, dendritic cells (DCs), and natural killer (NK) cells, were selected for characterization due to their significant involvement in RA pathogenesis.^[^ ^26^ ^]^ As shown in Figure 4e and Figure S8 (Supporting Information), compared to the HA group, the H/CS‐MOS hydrogel significantly enhanced cell recruitment due to the effect of MOS. Specifically, the number of macrophages increased by 2.87 fold, while monocytes increased by 1.30 fold, likely because monocytes serve as progenitors of macrophages and also express CD206. The numbers of other cells remained largely unchanged. In terms of cellular composition, macrophages, monocytes, and neutrophils collectively accounted for ≈78.77% (HA group) to 88.11% (H/CS‐MOS group) of the total recruited cells, leading to a focus on these three cell types in subsequent EV‐related investigations.

To investigate the effect of hydrogels on EV secretion, EVs were collected and quantified by NTA after a 4‐day co‐culture of different cell types with the hydrogels. As illustrated in Figure 4f and H/CS‐MOS hydrogels significantly enhanced EV secretion from macrophages, reaching 5.11 fold that of the no‐hydrogel group and 4.63 fold that of the HA group, with a markedly higher EV production than other cell groups. CD206‐expressing monocytes also exhibited a moderate increase in EV release, reaching 1.91 fold that of the no‐hydrogel group and 1.79 fold that of the HA group, whereas the neutrophil‐derived EV secretion remained largely unchanged. Collectively, these findings indicate that H/CS‐MOS hydrogels not only promote macrophage recruitment but also enhance their EV secretion capacity. Overall, considering both the proportion of cell types and the EV secretion levels, macrophage‐derived EVs are the primary contributors to the increased EV levels observed in vivo under H/CS‐MOS hydrogel stimulation. Further investigation examined the reparative effects of EVs derived from macrophages, monocytes, and neutrophils on RA chondrocytes after seven days of co‐culture. Western blot analysis (Figure 4g,h) revealed that EVs derived from macrophages co‐cultured with the H/CS‐MOS hydrogel significantly upregulated Col II expression while downregulating the expression of the catabolic marker MMP13. In future studies, more precise identification of EV cellular origins will be conducted using advanced and sophisticated techniques such as nanoflow cytometry and EV sequencing.

**Figure 5:**
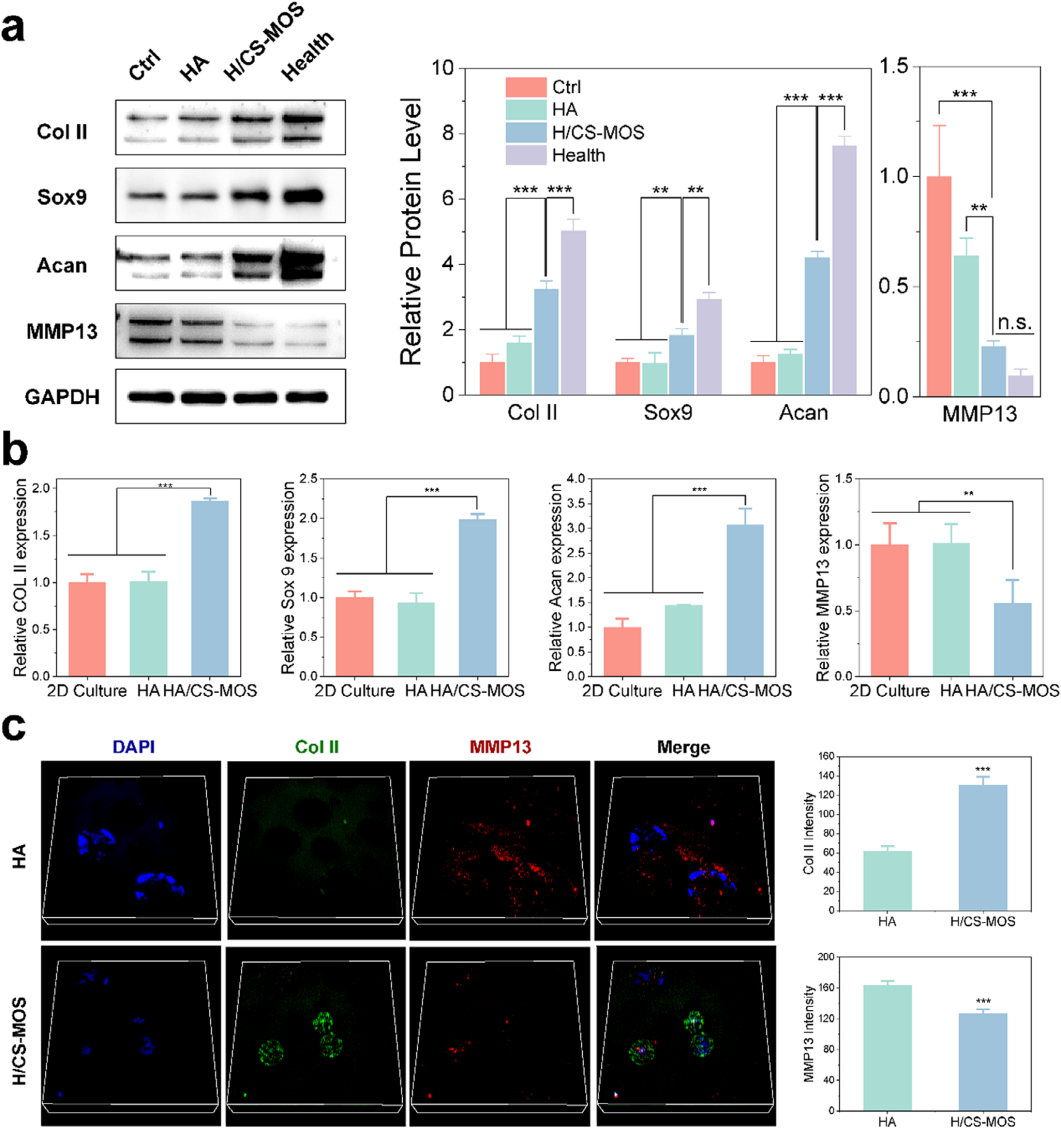
EVs from cells recruited in vivo by H/CS‐MOS hydrogel enhance RA cartilage repair. a) Protein levels of Col II, Sox9, Acan, and MMP13 in RA chondrocytes co‐cultured with EVs on the respective scaffolds, with corresponding quantification normalized to the control group (*n* = 3). b) mRNA levels of Col II, Sox 9, Acan, and MMP13, shown by RT‐PCR, with data normalized to the 2D Culture group (*n* = 3). c) Immunofluorescence and quantification of Col II and MMP13 on the scaffolds (*n* = 3). All data are presented as mean ± SD. (The *p* values were determined using unpaired two‐tailed t tests for two‐group comparisons and one‐way ANOVA followed by Tukey's post hoc test for multiple‐group comparisons. ^**^ *p* < 0.01, ^***^ *p* < 0.001, “n.s.”: no significant difference).

Since all recruited cells within the hydrogel function as an integrated entity, and the potential for crosstalk may occur among different cell types, further investigation was conducted to evaluate the effects of mixed EVs derived from the in vivo‐recruited heterogeneous cell population on RA chondrocytes. Western blot analysis of RA chondrocytes co‐cultured with mixed EVs on hydrogels revealed that EVs from the H/CS‐MOS group significantly upregulated the expression of chondrocyte marker proteins Col II, SOX9, and ACAN, while markedly downregulating the expression of the MMP13 protein (**Figure** **5**a). PCR analysis supported these findings at the gene level (Figure 5b). Immunofluorescent staining for chondrocyte markers Col II and the cartilage degradation marker MMP13 was performed to assess the influence of EVs on chondrocyte phenotype and distribution. As shown in Figure 5c, EVs from the H/CS‐MOS group significantly enhanced Col II expression in chondrocytes and reduced MMP13 levels. Additionally, the chondrocytes were observed to localize within the spherically formed pores created by the gas release, emphasizing the importance of hydrogel porosity.

### 2.6 In Vivo Immunomodulatory and Cartilage Repair Abilities of Hydrogels in a CIA Mouse Model

**Figure 6:**
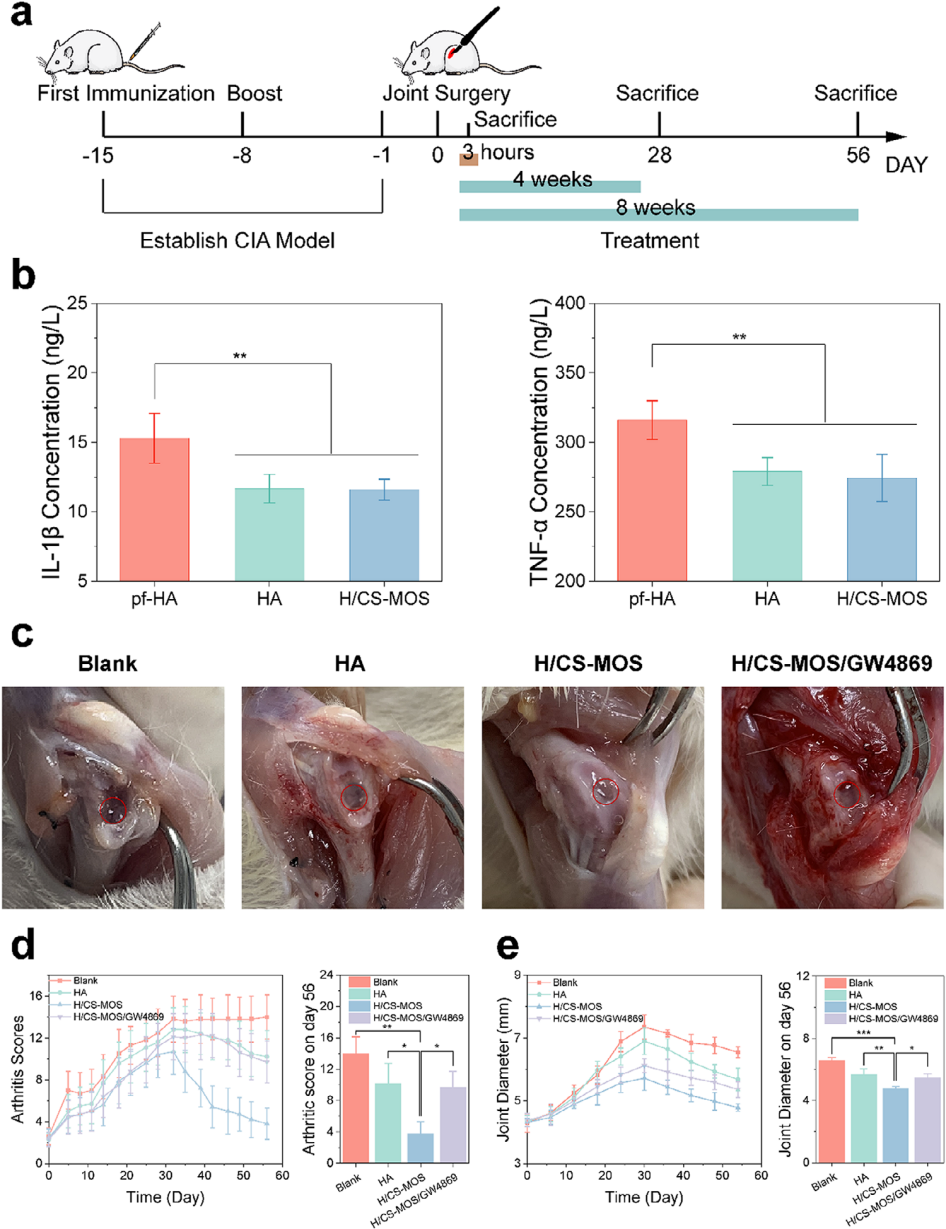
Therapeutic action of the H/CS‐MOS scaffold on full‐thickness joint defects in RA rats. a) Schematic illustration showing construction of the joint defect model in CIA mice and scaffold‐based treatment. b) Quantitative measurements of pro‐inflammatory (IL‐1β, TNF‐α) in the short‐term experiment. (pf‐HA: pre‐formed HA hydrogel) (*n* = 6). c) Representative macroscopic images of tissue regeneration at 12 weeks post‐surgery. d,e) Arthritis scores (d), and clinical joint diameters (e) of CIA mice that received different treatments throughout the treatment period, with statistical analysis on day 56 (*n* = 6). All data are presented as mean ± SD. (The *p* values were determined using two‐tailed one‐way ANOVA with a Tukey post hoc test. ^*^ *p* < 0.05, ^**^ *p* < 0.01, ^***^ *p* < 0.001).

In vitro experiments have demonstrated that hydrogels containing MOS can regulate inflammatory macrophages and promote the repair of damaged chondrocytes. To determine the hydrogel's potential for cartilage repair in an in vivo RA inflammatory environment, animal studies were conducted using a CIA model. As depicted in **Figure** **6**a, the CIA model was established in rats over the first two weeks. Full‐thickness osteochondral defects were then created in the femoral trochlear groove. A mixture of cross‐linking and polymer solutions was injected into the articular cavity after wound closure. In one experimental group of the long‐term study, GW4869, an EV inhibitor that blocks EV secretion, was incorporated into the hydrogel.^[^ ^27^ ^]^

A 3 h short‐term experiment was conducted to evaluate the impact of in situ carbon dioxide release during hydrogel crosslinking on the inflammatory microenvironment in arthritis. ELISA analysis showed that in comparison to the direct implantation of pre‐formed HA hydrogel, both in situ crosslinked HA and H/CS‐MOS hydrogels significantly reduced the levels of pro‐inflammatory cytokines IL‐1β and TNF‐α at the joint defect site (Figure 6b). These findings suggest that in situ carbon dioxide release plays a beneficial role in modulating inflammation.

For the long‐term experiment, macroscopic evaluation at 28 and 56 days post‐surgery revealed that cartilage defects in the RA environment were more challenging to repair compared to typical cartilage injuries (Figure 6c). In the blank, HA, and the hydrogel groups containing GW4869, the defects remained large, with noticeable negative effects on surrounding healthy tissue, such as visible wear and discoloration. In contrast, the H/CS‐MOS group exhibited substantial improvement, with the cartilage defects partially filled and minimal damage to the surrounding tissue.

Joint swelling, a key indicator of RA progression, was also assessed. As shown in Figure 6d,e, in the scaffold groups, both the arthritis index and joint diameter peaked at day 28 and gradually decreased thereafter, indicating inflammation resolution. However, in the blank group, the arthritis index continued to rise after day 28, and the joint diameter showed minimal reduction, suggesting persistent joint swelling and severe inflammation. In the HA and hydrogel groups with GW4869, only slight improvements were observed in the later stages. In contrast, the H/CS‐MOS group demonstrated a significant reduction in joint diameter by day 56, with the arthritis index dropping below 8. These results indicate that the MOS‐containing hydrogel was the most effective in alleviating joint swelling and reducing inflammation. By leveraging the early‐stage anti‐inflammatory effect of carbon dioxide, the chemical integration of MOS into the polymer matrix allowed the hydrogel to maintain its anti‐inflammatory effects throughout the entire experimental period.

**Figure 7:**
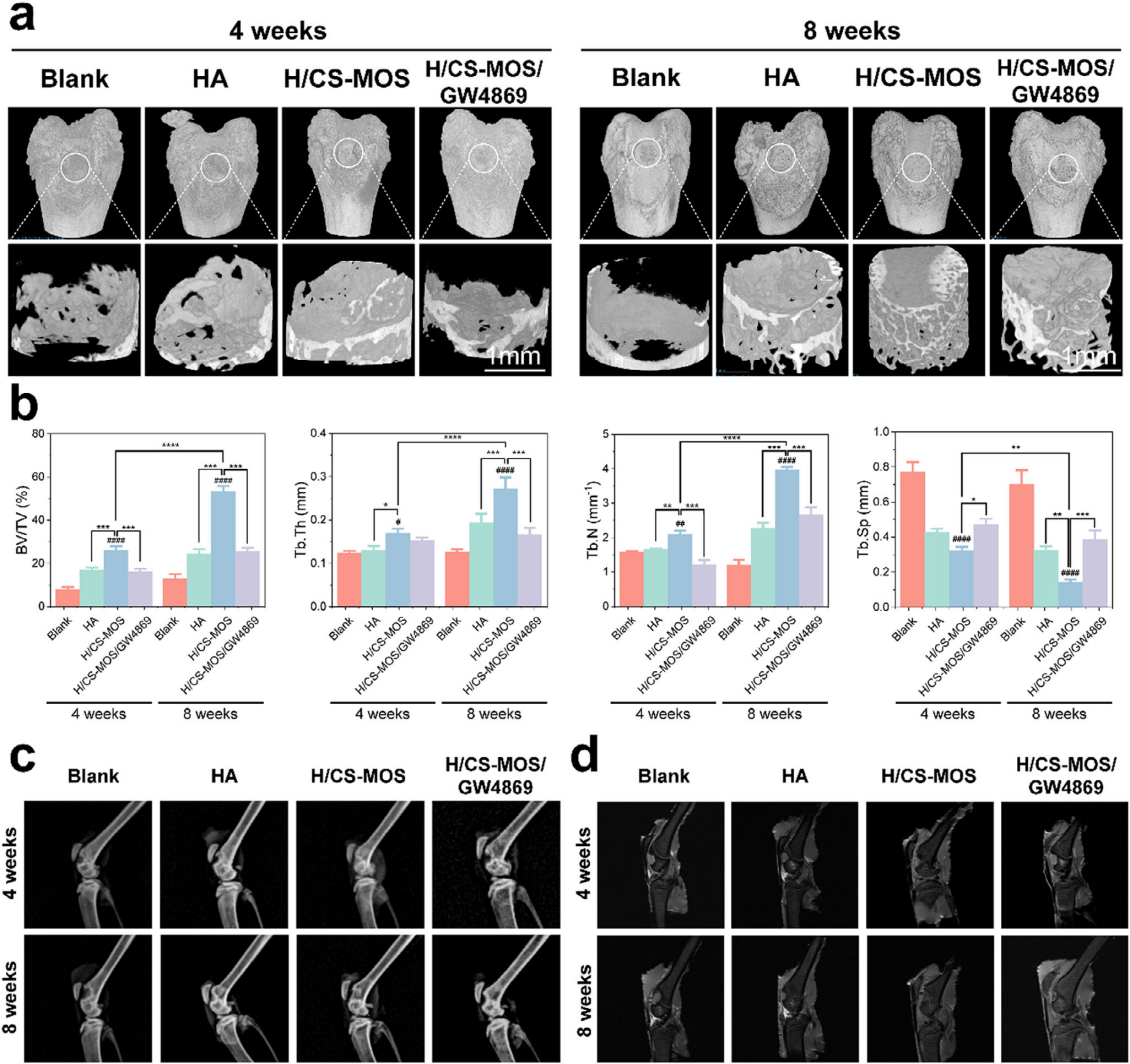
Assessment of repaired tissues in each group was conducted using micro‐CT, X‐ray, and MRI imaging techniques. a) Representative micro‐CT images of newly formed bone at 4 and 8 weeks across the different groups. b) Morphometric analysis quantified the volume of new bone within defect areas (*n* = 6). The parameters assessed include BV/TV, Tb.Th, Tb.N, and Tb.Sp. c,d) Representative images of X‐ray (c) and MRI (d) showing tissue regeneration at 4 and 8 weeks post‐surgery for each group. All data are presented as mean ± SD. (The *p* values were determined using two‐tailed one‐way ANOVA with a Tukey post hoc test. ^*^ *p* < 0.05, ^**^ *p* < 0.01, ^***^ *p* < 0.001, ^****^ *p* < 0.0001, ^#^ *p* < 0.05, ^##^ *p* < 0.01, ^####^ *p* < 0.0001, # indicates a comparison with the Blank group at their respective time points.

To evaluate subchondral bone regeneration, micro‐CT imaging was performed. Subchondral bone, being highly vascularized and nutrient‐rich, has a greater regenerative capacity compared to cartilage.^[^ ^28^ ^]^ At the 4‐week mark, when inflammation was at its peak, minimal bone formation was observed across all groups (**Figure** **7**a). In the blank group, where the absence of hydrogel led to significant cartilage loss and wear, almost no new bone formation was detected. In contrast, the H/CS‐MOS group exhibited slightly more bone regeneration than both the HA group and the hydrogel group containing GW4869. By 8 weeks, the blank group continued to show minimal bone growth, likely due to accelerated bone resorption driven by ongoing inflammation and cartilage degradation, which hindered new bone formation. In contrast, all other groups exhibited significant improvements in bone regeneration compared to the 4‐week time point. The H/CS‐MOS group displayed the most substantial bone growth, with new bone nearly filling the femoral defect edges and extending into the defect itself. As shown in Figure 7b, quantitative micro‐CT analysis revealed that the H/CS‐MOS group had a significantly higher bone volume‐to‐total volume (BV/TV) ratio compared to the HA group, the GW4869‐containing hydrogel group, and far exceeding the blank group. Bone growth in the H/CS‐MOS group was 2.21 times greater than in the HA group, 2.16 times higher than in the GW4869 group, and 5.09 times more than in the blank group. Additionally, the H/CS‐MOS group exhibited higher values for trabecular thickness (Tb.Th), trabecular number (Tb.N), and bone mineral density (BMD), while trabecular separation (Tb.Sp) was lower, indicating a denser and more mature mineralized matrix in the defect area. These findings suggest that the MOS‐containing hydrogel not only regulates inflammation but also significantly enhances bone repair and regeneration.

X‐ray and MRI analyses were conducted to further evaluate defect repair (Figure 7c,d). The imaging results revealed large voids in the defects of the blank, HA, and GW4869 hydrogel groups, which were consistent with macroscopic observations. At 8 weeks, the defect size in the blank group had increased compared to the 4‐week mark, indicating worsening cartilage damage. In the HA and GW4869 hydrogel groups, the defects were partially filled with newly formed bone and cartilage tissue, although noticeable depressions persisted. Remarkably, in the H/CS‐MOS group, the defects were nearly filled, resulting in a smoother and more even surface. A comparison between groups with and without GW4869 further underscores the critical role of EVs secreted by regulated macrophages. After macrophage polarization, EVs play a role in sending signals to chondrocytes and other cells, modulating the inflammatory environment and promoting tissue repair. Complete characterization of the structure and composition of the newly formed tissues will require further detailed analysis.

**Figure 8:**
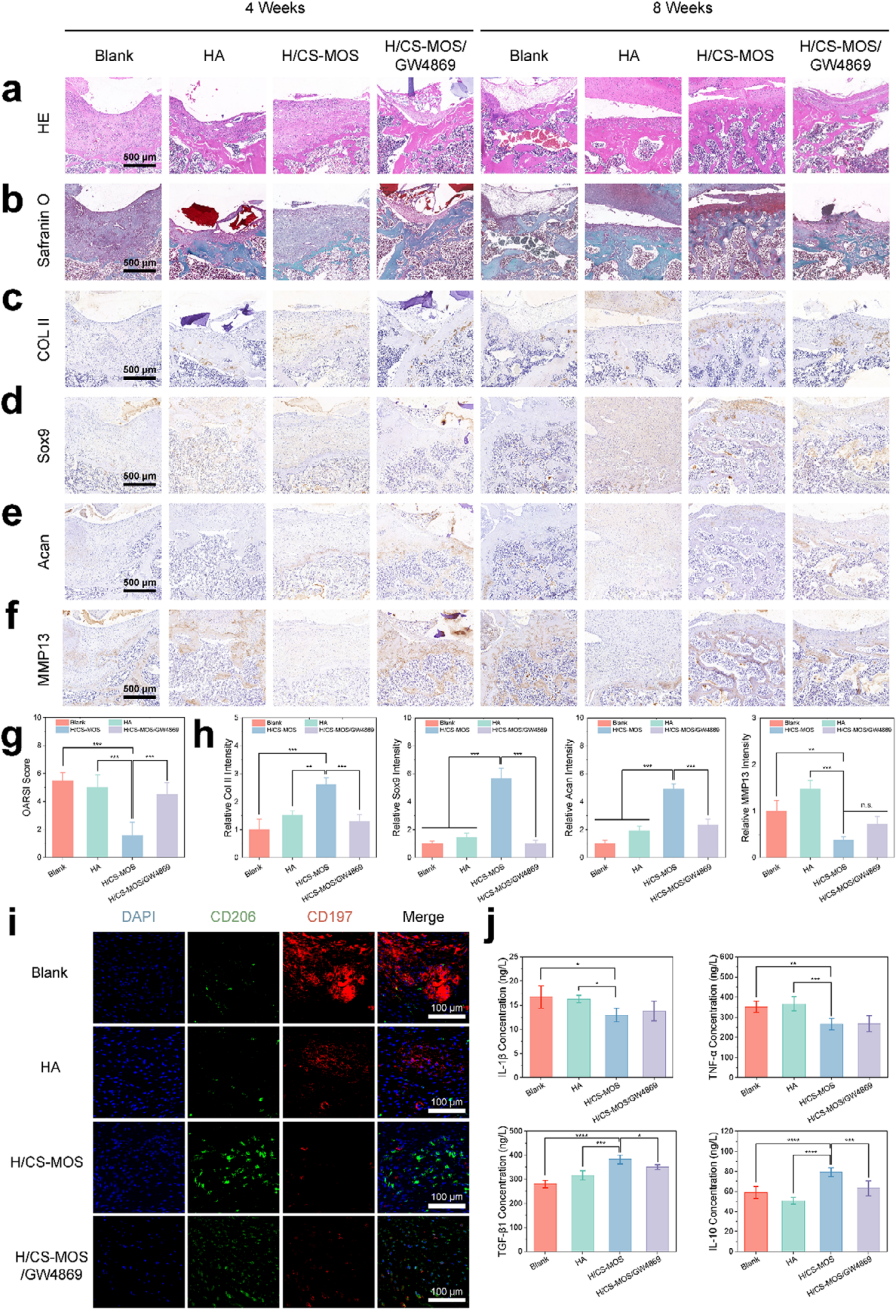
Histological and immunohistochemical assessments of cartilage regeneration were conducted at 4 and 8 weeks after surgery. a,b) H&E (a) and Safranin‐O/Fast Green staining (b) of tissue sections surrounding the defects. c–f) Immunohistochemical staining of collagen type II (c), Sox9 (d), Acan (e), and MMP13 (f) in the areas surrounding the defects. g) OARSI scoring system for the histological assessment (*n* = 6). h) Quantitative analysis of immunohistochemical staining for collagen type II, Sox9, Acan, and MMP13 at 8 weeks, with data normalized to the blank group (*n* = 6). i) Immunofluorescence staining images of CD197 and CD206 (green: CD197; red: CD206; blue: DAPI). j) Quantitative measurements of pro‐inflammatory (IL‐1β, TNF‐α) and anti‐inflammatory (TGF‐β1) cytokines in rat sera (*n* = 6). All data are presented as mean ± SD. (The *p* values were determined using two‐tailed one‐way ANOVA with a Tukey post hoc test. ^*^ *p* < 0.05, ^**^ *p* < 0.01, ^***^ *p* < 0.001, ^****^ *p* < 0.0001, “n.s.”: no significant difference).

Building on the imaging findings, the recovery of defect sites was further evaluated through HE, Safranin O/Fast Green, Toluidine Blue, and immunohistochemical staining. At 4 weeks, hydrogel remnants were still present in the defects, with new tissue forming and integrating into the hydrogel's porous structure. By 8 weeks, all groups showed complete hydrogel degradation and absorption, with no remnants observed. HE staining (**Figure** **8**a) revealed extensive inflammatory cell infiltration in the blank and HA groups, which worsened over time. By 8 weeks, the blank group displayed loose, edematous fibrous tissue, a condition that could accelerate cartilage deterioration through erosion and degeneration.^[^ ^2a^ ^]^ In contrast, the groups treated with MOS exhibited reduced inflammatory cell infiltration by 8 weeks, emphasizing the anti‐inflammatory properties of MOS. This reduction in inflammation was further corroborated by iNOS staining (Figure S9, Supporting Information), which showed a significant decrease in iNOS expression in the H/CS‐MOS group at 8 weeks, regardless of the addition of GW4869. Histological evaluation with Safranin O/Fast Green (Figure 8b) and Toluidine Blue staining (Figure S10, Supporting Information) revealed that the blank group exhibited a rough cartilage surface, vertical fissures, and limited cartilage regeneration by 8 weeks. Similarly, minimal signs of repair were observed in the HA group and the hydrogel group treated with GW4869. Conversely, the H/CS‐MOS group showed a smoother cartilage surface, increased cartilage thickness, and enhanced extracellular matrix deposition, resulting in a significantly lower OARSI score (Figure 8g). Immunohistochemical staining (Figure 8c–f) and corresponding quantitative analysis (Figure 8h) further confirmed that the H/CS‐MOS group exhibited significantly increased expression of cartilage‐specific markers—Col II, Sox9, and Acan—alongside a substantial reduction in the cartilage catabolic enzyme MMP13, when compared with other groups. Collectively, these results demonstrate that only the H/CS‐MOS group achieved significant cartilage regeneration at the defect site. Notably, the hydrogel group co‐treated with GW4869 exhibited minimal cartilage formation, suggesting that effective cartilage repair relies on EV‐mediated signaling within a properly regulated inflammatory microenvironment to exert therapeutic effects on damaged chondrocytes.

To further investigate the inflammatory response at the defect sites, immunofluorescent staining was used to analyze markers of M1 and M2 macrophages. As shown in Figure 8i, the blank and HA groups predominantly displayed M1 macrophages, with the highest density observed in the blank group. Conversely, the hydrogel groups containing MOS showed a pronounced increase in M2 macrophages, with the H/CS‐MOS group exhibiting the highest M2/M1 ratio, highlighting the role of MOS in promoting macrophage polarization toward the M2 phenotype. In the GW4869 group, the limited presence of M2 macrophages was attributed to poor cartilage repair, suggesting that EV inhibition impaired tissue regeneration. This imbalance likely favored the M1 pro‐inflammatory phenotype, resulting in a mixed presence of both macrophage populations in the GW4869 group.

Given that RA is an autoimmune disease driven by inflammatory cytokines, the concentrations of both pro‐inflammatory (IL‐1β, TNF‐α) and anti‐inflammatory (IL‐10, TGF‐β1) cytokines at the defect were determined. As shown in Figure 8j, the blank and HA groups exhibited significantly elevated pro‐inflammatory cytokines and reduced anti‐inflammatory cytokines relative to the MOS‐containing hydrogel groups. The GW4869 group displayed lower anti‐inflammatory cytokine levels than the MOS group without the inhibitor, further highlighting the critical role of EV signaling in modulating inflammation. These findings demonstrate that the MOS‐containing hydrogel effectively regulates the inflammatory environment in the RA model by promoting M2 macrophage polarization and reducing inflammation. Furthermore, EVs act as crucial mediators between macrophages and chondrocytes, playing an indispensable role in tissue repair and RA treatment.

## 3 Conclusion

In summary, an injectable hydrogel capable of in situ pore formation was designed to modulate macrophage polarization and regulate the inflammatory environment at joint sites. The hydrogel achieves in situ pore formation through a controlled process of simultaneous cross‐linking and gas generation, integrating both injectability and porosity into a single material. With a 30 s injection window and ≈86% porosity, it meets clinical requirements for minimally invasive treatments while providing a 3D platform that accelerates cell growth and repair within the defect.

The chemical grafting of MOS groups onto the polymer chains enables the hydrogel to regulate macrophage behavior and the EVs they secrete over the long term. In vitro experiments demonstrated that the MOS‐containing hydrogel effectively recruits macrophages, particularly those of the M2 phenotype, and induces M1 to M2 macrophage polarization. The EVs secreted by these M2 macrophages are crucial for chondrocyte repair and cartilage matrix production.

In vivo experiments further confirmed that the MOS‐incorporated hydrogel alleviates inflammation through initial carbon dioxide release and sustained regulation by MOS, while promoting cartilage matrix regeneration in the RA model. Notably, the EV inhibitor‐treated group failed to generate stable and healthy cartilage, highlighting the critical role of EVs in mediating intercellular communication. These findings not only present a novel injectable hydrogel with in situ pore formation as a tissue engineering scaffold, but also offer a straightforward and more cost‐effective RA treatment. This development marks a promising advancement in RA clinical therapy and lays a solid foundation for future therapeutic innovations.

## 4 Experimental Section

### Materials

The details of the purchase of chemicals are as follows. Hyaluronic acid (HA, Yuanye, Shanghai, China), Cystamine dihydrochloride (Aladin, Shanghai, China), 1‐(3‐Dimethylaminopropyl)‐3‐ethylcarbodiimide hydrochloride (EDC, Aladin), N‐hydroxysuccinimide (NHS, Aladin), Chondroitin sulfate (CS, Aladin), Sodium periodate (NaIO_4,_ Aladin), Adipic dihydrazide (ADH, Aladin), Sodium cyanoborohydride (NaBH₃CN, Aladin), Mannose oligosaccharide (MOS, Solarbio, Beijing, China), M‐CSF (Protech, Santa Clara, CA, USA), Penicillin and streptomycin (Gibco, Waltham, MA, USA), α‐MEM (Gibco), DMEM (Gibco), DMEM/F12 (Gibco), Fetal Bovine Serum (FBS, Gibco), bovine type II collagen (chondrex, Woodinville, WA, USA) and complete Freund's adjuvant (CFA, chondrex). All chemicals were of analytical grade and were not purified further.

### Hydrogel Formation

a) Synthesis of Aldehyde‐Modified Mannose Oligosaccharide (OxMOS)

Mannose oligosaccharide (MOS) was first dissolved in ultrapure water. In a separate container, Sodium periodate (NaIO_4_) at a concentration of 2 molar equivalents was solubilized in ultrapure water, ensuring the solution was protected from light, and was added dropwise to the MOS solution with stirring in the dark at 25 °C for 16 h to complete the oxidation reaction. Afterward, the reaction mixture underwent dialysis against ultrapure water for 2 days with a 500 Da‐cutoff membrane (Spectrum Medical, Chelmsford, MA, USA). The final product, oxidized MOS (OxMOS), was obtained by vacuum freeze‐drying the dialyzed solution.

b) Preparation of mannose oligosaccharide‐adipic dihydrazide (MOS‐ADH)

To prepare the solution, 0.01 mol of adipic dihydrazide (ADH) was thoroughly dissolved in ultrapure water. Next, a slow dropwise addition of OxMOS (0.1 g), also dissolved in ultrapure water, was made to the ADH solution. The reaction was continued for 2 h at room temperature (RT). Next, 0.01 mol of sodium cyanoborohydride (NaBH₃CN) was added in three portions, and the reaction was continued with stirring for 24 h at RT. After the conclusion of the reaction, the material underwent dialysis against ultrapure water for 2 days using a 500 Da cutoff membrane. Finally, the purified mannose MOS‐ADH was obtained by removing water through vacuum freeze‐drying.

c) Preparation of Chondroitin Sulfate‐Mannose Oligosaccharide (CS‐MOS)

Chondroitin sulfate (CS) (100 mg) was dissolved in 10 mL of PBS (pH 5.5, Beyotime, Shanghai, China) with continuous stirring until fully dissolved. Afterward, EDC and NHS were introduced for 1 h to activate the carboxyl groups. Once activated, MOS‐ADH (500 mg) was supplemented into the mixture, which was then agitated at RT for 24 h. This was followed by dialysis against ultrapure water for 2 days using a 7000 Da cutoff membrane (Spectrum Labs). The final product, CS‐MOS, was obtained by vacuum freeze‐drying for 48 h.

d) Preparation of H/CS‐MOS Hydrogel

At room temperature, various amounts of CS‐MOS were dissolved in ultrapure water. Hyaluronic acid (HA) was subsequently added to the CS‐MOS solution and mixed thoroughly to ensure dissolution. For H‐0.1CM, the ratio of HA to CS‐MOS was 10:1, while for H‐0.2CM (H/CS‐MOS), the ratio was 5:1. Cystamine dihydrochloride was then added at a 2:1 molar ratio between the carboxyl moieties of HA and the amino moieties of the crosslinker, and the mixture was well combined. This was followed by the successive addition of NHS and EDC in a 2:3 molar ratio relative to the carboxyl groups of HA, stirring the solution to activate the carboxyl groups and facilitate the amide reaction. The mixture was then incubated at 37 °C to allow gelation. The preparation of the pure HA hydrogel followed the same steps, without the addition of CS‐MOS.

### Chemical Structure Analysis

The chemical structures of OxMOS, MOS‐ADH, and CS‐MOS were analyzed qualitatively using FTIR spectroscopy (IS41 NICOLET, Thermo Fisher, Waltham, MA, USA), covering a spectral range of 4000 to 400 cm¹. In addition, the chemical structures of MOS‐ADH and CS‐MOS were further characterized by ¹H‐NMR spectroscopy (AVANCE III, Bruker, Germany), recording spectra at a frequency of 600 MHz.

### Rheological Studies

The rheological characteristics of the H/CS‐MOS hydrogel were evaluated using a rotational rheometer (RS600, Thermo Hakke, Karlsruhe, Germany).

(a) Using a constant frequency of 1 rad s^−1^, a time sweep was conducted at 37 °C to monitor the gelation process. The hydrogel precursor solution was applied to a parallel plate (20 mm), and the storage (G′) and loss (G″) moduli were measured over 15 min to track changes during gel formation.

(b) Using a fixed strain of 1%, a frequency sweep from 0.1 to 10 rad s^−1^ was carried out at 37 °C to assess the variation of G' with frequency for the H/CS‐MOS hydrogel.

### Observation of the Micromorphology

The micromorphology of the H/CS‐MOS hydrogel was analyzed using various imaging techniques. Non‐lyophilized samples with in situ generated porous structures were observed using micro‐computed tomography (Micro‐CT, Skyscan 1172, Bruker), inverted microscope (TE2000U, Nikon, Tokyo, Japan), and Cryo‐SEM (JSM‐6700F, JEOL, Tokyo, Japan). For lyophilized samples, the porous morphology was observed using SEM (S‐4800, Hitachi, Tokyo, Japan). To quantify the pore size and porosity, structural parameters were calculated from the Micro‐CT images.

### Mechanical Studies

The compression characteristics of the hydrogels were evaluated using a universal testing machine (AG‐2000A, Shimadzu, Kyoto, Japan) to assess the effect of CS‐MOS incorporation and its concentration on compressive performance. Samples, cylindrically shaped, and having a height and a radius of 8 mm and 5 mm respectively, were subjected to a compression strain rate of 5 mm min^−1^. The compressive modulus and compressive strength were determined from the resulting stress–strain curves.

### Swelling Test

An in vitro swelling study of the hydrogels was carried out in PBS (Beyotime), pH 7.4. Pre‐lyophilized hydrogel samples were weighed to record the dry weight (*W_dry_*). The specimens were then submerged in PBS at a ratio of 1:10 (dry weight to solution volume) and gently shaken in a 37 °C incubator. At specific intervals of 1, 4, 7, 10, 14, and 21 days, the hydrogels were removed, gently blotted to eliminate excess surface moisture, and reweighed to record their swollen weight (*W_wet_*). The swelling ratio was determined as:

(1) $$Swelling\;ratio=\frac{W_{wet}-W_{dry}}{W_{dry}}\times 100\%$$

### In Vitro Degradation Test

The in vitro degradation experiment of the H/CS‐MOS hydrogel was conducted using Tris‐HCl buffer (Beyotime) with a pH of 7.4. The initial dry weight (*W_dry_*) of the hydrogel samples was noted following pre‐liophilization. The hydrogel samples were then immersed in Tris‐HCl buffer at a ratio of 1:10 (dry hydrogel weight to solution volume) and gently agitated in a 37 °C incubator shaker. At predetermined time points of 1, 4, 7, 10, 14, and 21 days, the samples were removed, lyophilized, and weighed to record the dry weight at each time point (*W_d_*). The degradation rate of the hydrogel was determined as follows:

(2) $$Degradation\;\;rate=\frac{W_{dry}-W_{d}}{W_{dry}}\times 100\%$$

### Subcutaneous Implantation Model in the Dorsal Region of Mice

The animal experiments in this study were approved by the Animal Research Committee of Shanghai Jiao Tong University Affiliated Sixth People's Hospital, in compliance with the established “3Rs” principles. To investigate the regulation of protein expression in cells interacting with the H/CS‐MOS hydrogel in vivo, C57BL/6 mice were employed as a subcutaneous implantation model. The procedure was as follows:

Male C57BL/6 mice, weighing between 20–22 g, were first anesthetized. Once the mice were under deep anesthesia, their dorsal fur was shaved with a clipper, and the surgical area was disinfected with alcohol swabs. A small incision was made in the shaved area using surgical scissors, and the skin was carefully separated from the underlying fascia. Sterile hydrogel samples, each with 1 mm thickness and 5 mm diameter, were then implanted subcutaneously through the incision. The incision was sutured, and the mice were allowed to recover and resume normal feeding. The mice were euthanized on the fourth day, and the implanted samples were retrieved for subsequent analysis.

### Analysis of the Nature and Quantity of In Vivo Recruited Cells

The in vivo recruitment of macrophages was assessed using the macrophage surface antigen F4/80 and FITC‐Phalloidin. CD44 and CD206 expression levels were analyzed to determine whether the H/CS‐MOS hydrogel enhances protein expression in cells adhering to its surface. Four days after the subcutaneous implantation of the hydrogels in mice, the implants were retrieved and fixed with glutaraldehyde for further analysis. The samples were treated (overnight, 4 °C) with anti‐F4/80 (ab300421, Abcam, Cambridge, UK) and anti‐CD44 (ab316123, Abcam)/anti‐CD206 (ab64693, Abcam) antibodies. Phalloidin (Sigma–Aldrich, St. Louis, MO, USA) was used to satin the cytoskeleton and DAPI (Sigma‐Aldrich) was used for nuclear staining. Fluorescence images were subsequently observed using CLSM (A1R, Nikon).

Cells on the surface of the in vivo implanted hydrogels were analyzed by flow cytometry. Macrophages were identified using CD11b and F4/80 antibodies (BD, Franklin Lakes, NJ, USA). Surface proteins were labeled with anti‐CD44 and anti‐CD206 antibodies (BioLegend, San Diego, CA, USA). Macrophage polarization was assessed using the M1 marker CD197 and the M2 marker CD206. Four days after the subcutaneous implantation of hydrogels in mice, the materials were retrieved, and the surface cells were acquired by enzymatic digestion with a mixed enzyme solution (50% trypsin (Thermo Fisher) + 50% collagenase (Worthington, Lakewood, NJ, USA)). After blocking, the cells were allowed to incubate with the specific antibodies. Flow cytometry was then conducted using a C6 LSRII flow cytometer (BD) to analyze the cells.

### Extraction and Isolation of Mouse Bone Marrow Monocytes (BMM)

C57BL/6 mice, weighing 20–22 g, were sacrificed by cervical dislocation and then submerged in 75% ethanol (Titansci, Shanghai, China) for 10 min. The femurs and tibias were isolated, and the epiphyses were removed with surgical scissors to access the bone marrow cavities at both ends. These cavities were flushed multiple times with α‐MEM medium containing 15% FBS using a syringe. The collected bone marrow solution was centrifuged (1000 rpm, 5 min, RT), discarding the supernatant. The precipitate was resuspended in α‐MEM with 10% FBS and shifted to a sterile 6 cm cell culture dish, wherein 5 ng mL^−1^ M‐CSF was added to it. After incubation of the cells at 37 °C for 24 h, the supernatant was filtered through a 40 µm sieve and centrifuged (800 rpm, 4 min). A certain volume (4 mL) of complete α‐MEM with 10% FBS and 50 ng mL^−1^ M‐CSF was used to resuspend the cell pellet, and incubation was continued at 37 °C for an additional 48 h, followed by washing with PBS. Digestion was performed by adding 1 mL of Versene (Gibco) for 10 min. Subsequently, the addition of 2 mL of medium terminated the digestion, the solution was centrifuged (800 rpm, 4 min). The pelleted cells were resuspended and cultured in α‐MEM with 10% FBS.

### Anti‐Inflammatory Test

Prior to seeding onto the hydrogel, BMM cells underwent M1 polarization. Cells (1 × 10^6^ mL^−1^) were inoculated in 6‐well plates and grown in DMEM with 10% FBS, supplemented with 500 ng mL^−1^ LPS (Sigma–Aldrich) for 2 h. Following this initial stimulation, the medium was replaced with fresh DMEM containing 10% FBS and 20 ng mL^−1^ IFN‐γ (BioLegend) and the cells were grown for an additional 24 h to promote M1 polarization.

After this induction period, ≈1 million M1‐polarized macrophages were seeded onto HA and H/CS‐MOS hydrogels in a 24‐well plate and incubated for 48 h. The incubation was followed by careful removal of the medium and gentle washing of the cells with PBS. The hydrogels were finely chopped using surgical scissors and covered with phenol red‐free trypsin (Thermo Fisher). The mixture was subjected to a 10 min incubation at 37 °C to allow dissociation of the cells. The cell suspension was collected, and centrifuged at 1000 rpm for 5 min at 4 °C, following which the supernatant was disposed of. The leftover cell pellet was added to 200 µL of cell staining buffer (BioLegend) for blocking. Cells were then stained with CD197 and CD206 antibodies and analyzed using flow cytometry to evaluate the polarization markers.

Besides the evaluation of macrophage polarization through flow cytometry, inflammatory factor levels in hydrogel‐adherent cells were examined by Western blotting. After finely chopping the hydrogel, the cells were lysed with RIPA buffer with 1% PMSF (Beyotime). Protein concentrations were quantified using a BCA assay kit (Beyotime) and adjusted to ensure equal amounts across samples. Next, a 5× loading buffer was added to the protein specimens with heating at 100 °C for 5 min for denaturation. The specimens were stored at −20 °C until subsequent use. For electrophoresis, 20 µL of individual specimens were loaded onto a 15% SDS‐PAGE gel (Beyotime), and electrophoresis was conducted initially at 80 V and then at 100 V. Following this, proteins were transferred to a methanol‐activated PVDF membrane (Thermo Fisher) at 300 mA for 70 min. A blocking buffer was used to block the membrane, which was then cut according to the corresponding Kd values for subsequent analysis. The membrane pieces were probed overnight at 4 °C with primary antibodies against GAPDH (#2118, Cell Signaling Technology, CST, Danvers, MA, USA), CD206 (ab64693, Abcam), CD44 (ab157107, Abcam), IFN‐γ (ab198801, Abcam), TNF‐α (ab307164, Abcam), IL‐1Ra (NBP1‐32568, Novus Biologicals, Littleton, CO, USA) and Arg‐1 (#93668, CST). Following the removal of primary antibodies, the membranes were rinsed three times with TBST (Beyotime). They were then incubated with HRP‐conjugated secondary antibodies (#7074, CST) at room temperature for 2 h. After this incubation, the membranes were washed three additional times with TBST solution. ECL chemiluminescent substrate (CST) was subsequently applied to detect the signals. These signals were then captured using a chemiluminescent imaging system (Tanon 5200, Tanon, Shanghai, China).

Furthermore, ELISA tests were performed to assess the levels of inflammation‐related cytokines in the cell culture supernatant. The concentrations of IL‐6, IL‐1β, TNF‐α, IL‐10, and TGF‐β in the supernatant were measured using the corresponding ELISA kits, following the manufacturer's instructions.

### Macrophage Metabolism Assays

The glucose metabolism levels of macrophages cultured with the scaffolds were assessed. M1‐polarized cells were seeded onto HA and H/CS‐MOS hydrogels in 6‐well plates, with cells cultured in only the medium serving as the control group. After 24 h of incubation, the supernatant from each group was collected, and glucose uptake was analyzed using the Glucose Assay Kit (#DIGL‐100, Bioassay Systems, CA, USA). Cells were lysed by ultrasonication (for pyruvate detection) or special lysis (for ATP detection), followed by centrifugation. The supernatant was then collected and analyzed for intracellular pyruvate and ATP production using the Pyruvate Assay Kit (#BC2205, Solarbio, Beijing, China) and the Enhanced ATP Assay Kit (#S0027, Beyotime, Shanghai, China), respectively. All assays were performed according to the manufacturer's instructions, and results were normalized to cell number.

The key pathways of macrophage glucose metabolism were characterized using RT‐PCR and Seahorse assays. mRNA levels of Glut‐1, PISD, and TFAM were analyzed using RT‐PCR. Total mRNA was extracted, reverse‐transcribed to cDNA using ReverTra Ace kits (Toyobo, Osaka, Japan) and amplified with a thermal cycler (Biometra, Göttingen, Germany) with the following settings: 37 °C for 15 min and 85 °C for 5 s. The collected samples were stored at −80 °C. The samples were subsequently analyzed using SYBR Premix Ex Taq kits (Takara Bio, Shiga. Japan) on a Bio‐Rad PCR instrument (Bio‐Rad, Hercules, CA, USA). A list of primer sequences is appended in Table S2 (Supporting Information). The oxygen consumption rate (OCR) and extracellular acidification rate (ECAR) of macrophages on different samples were analyzed using a Seahorse XF96 extracellular flux analyzer (Seahorse Bioscience, MA, USA). M1‐polarized cells were seeded onto HA and H/CS‐MOS hydrogels in 6‐well plates, with cells cultured in only the medium serving as the control group. Cells on the scaffolds were digested using the aforementioned method and replated at a density of 1.5 × 10⁴ cells per well. After an additional 24 h incubation, the culture medium was replaced with Seahorse assay medium, and the cells were incubated at 37 °C in a non‐CO₂ incubator for 60 min before OCR and ECAR quantification. For OCR analysis, 1 µm oligomycin (ATP synthase inhibitor), 1 µm carbonyl cyanide‐4‐(trifluoromethoxy) phenylhydrazone (FCCP, mitochondrial respiration uncoupling agent), and 0.5 µm rotenone & antimycin A (R&AA, inhibitors of complex I and III of the electron transport chain, respectively) were sequentially injected during real‐time OCR measurements. For ECAR analysis, 10 mM glucose, 1 µm oligomycin, and 50 mM 2‐deoxyglucose (2‐DG, glycolysis inhibitor) were sequentially injected during real‐time ECAR measurements.

Furthermore, the autophagy levels and expression of related signaling proteins in macrophages cultured on the scaffolds were analyzed using Western blot. The Western Blot procedure was similar to that previously described, with incubation using antibodies against LC3 (#12741, CST), p‐AMPK (#2535, CST), AMPK (#2532, CST), p‐mTOR (#5536, CST), mTOR (#2983, CST), and β‐actin (#3700, CST).

### Establishment of the RA Mouse Model

RA induction was performed in SPF‐grade male C57BL/6J mice with weights ranging from 20–22 g. A 2 mg mL^−1^ solution of bovine type II collagen in acetic acid was prepared and combined with an equal volume of complete Freund's adjuvant (CFA) using a glass syringe on ice. The mixture was emulsified by repeatedly drawing and expelling the solution through the syringe until a uniform emulsion was obtained. The mice were injected with 0.1 mL of the emulsion at the base of the tail, inserting the needle 2 cm from the base of the tail and injecting 0.5 cm from the tip of the needle. After 21 days, the mice received a booster injection of 0.1 mL of the emulsion at the base of the tail.

### Extraction and Isolation of Extracellular Vesicles (EVs)

After 4 days of injection of HA and H/CS‐MOS hydrogels in vivo, the materials were retrieved and placed in α‐MEM medium containing 2% EV‐free serum (Gibco). The samples were then incubated in a cell culture incubator at 37 °C with 5% CO₂. Once the EV concentration in the medium reached saturation, the culture medium was collected and filtered through a 220 nm membrane to allow the removal of larger cellular debris. The filtered solution was then subjected to ultracentrifugation (10 000 × g, 30 min, 4 °C) (Sorvall WX+, Thermo Fisher). Following careful collection of the supernatant, it was re‐centrifuged (100 000×g, 90 min, 4 °C). Most of the supernatant was removed, leaving ≈100 µL of liquid at the tube bottom. The bottom of the ultracentrifuge tube was washed multiple times to ensure complete recovery of the EVs.

### Detection and Characterization of EVs

a) A nanoparticle tracking analyzer (Zetaview, Particle Metrix, Meerbusch, Germany) was employed for measuring the concentration and particle size of the EVs. b) The morphological characteristics of the EVs were examined using TEM. Following careful deposition onto a copper grid, the EVs settled for 20 min. After removing the excess liquid, the EVs were stained with lead acetate solution for 1 min, with any surplus lead acetate solution carefully removed afterward. Following the complete drying of the samples, they were observed using TEM (JEM‐2100F, JEOL). Surface marker proteins were analyzed by Western Blot. EVs and cells from the HA and H/CS‐MOS groups were lysed using sonication to obtain the lysate. The control group (C group) comprised cells cultured under normal conditions. The Western Blot procedure was similar to that previously described, with incubation using antibodies against CD9 (ab223052, Abcam), CD63 (ab217345, Abcam), CD81 (ab109201, Abcam) and Calnexin (ab227310, Abcam).

### Extraction and Isolation of Chondrocytes from RA Mice

After inducing RA in the mice for ≈1 month, when visible joint swelling was observed, the C57BL/6 mice were euthanized by cervical dislocation and immersed in 75% ethanol for 5 min. The skin was then incised with a scalpel, and the joint capsule was carefully opened along the lower edge of the patella. The ligaments were severed, the knee joint disarticulated, and the surface connective tissue removed. Approximately half of the cartilage layer was shaved off with a scalpel and placed in a dish containing PBS with antibiotics (Thermo Fisher). The cartilage was cut into small pieces and transferred to a 15 mL centrifuge tube, where it was washed twice with PBS containing antibiotics. To digest the tissue, an amount of 0.25% trypsin, three times the tissue volume, was added, and incubated for 20 min. The digestion was halted by addition culture medium, followed by centrifugation (1000 rpm, 5 min). After discarding the supernatant, the tissue was resuspended in 0.2% type II collagenase (Worthington) for an additional 4 h of digestion in the incubator. After digestion, DMEM medium was added, and the mixture was pipetted thoroughly before being filtered through a 200‐mesh filter. The filtrate was collected and centrifuged at 1000 rpm for 5 min. After removal of the supernatant, the cells were grown in DMEM/F12 with 15% FBS.

### Effect of EVs from In Vivo Recruited Cells on the Viability of RA Chondrocytes

After co‐culturing RA chondrocytes with EVs from both the H and H/CS‐MOS groups at a concentration of 0.5 × 10⁹ mL⁻¹ for 3 days, chondrocyte proliferation was assessed by CCK‐8 assays, according to the provided protocol. Chondrocytes grown in DMEM/F12 medium without EV‐free serum were classified as the Ctrl‐group, while those grown in DMEM/F12 with FBS were categorized as the Ctrl+ group.

### Flow Cytometric Analysis of Cells Recruited by the Hydrogels

First, an RA mouse joint defect model was established. Once the disease had developed, mice were anesthetized with isoflurane. After trimming the fur and disinfecting the skin, a medial knee incision was made, and the joint capsule was carefully exposed. A full‐thickness osteochondral defect (0.8 mm deep, 1 mm in diameter) was created in the trochlear groove of the left femur using a microdrill. The incision was then sutured, and a polymer precursor solution with a crosslinking agent was injected into the joint cavity using a syringe.

Mice were sacrificed on the fourth day after hydrogel injection, and the hydrogels were retrieved. The samples were digested and processed as previously described. After blocking, macrophages were labeled with F4/80 and CD11b antibodies, monocytes and neutrophils were labeled with CD11b, LY6C, and LY6G antibodies (Biolegend), classical dendritic cells (cDC) were labeled with CD11c and MHC II antibodies (Biolegend), plasmacytoid dendritic cells (pDC) were labeled with Siglec‐H and CD317 antibodies (Biolegend), and NK cells were labeled with NK1.1 and CD49b antibodies (Biolegend). Flow cytometry was then conducted to analyze the cells.

### Effect of the Hydrogels on EV Secretion by Cells

M1‐induced macrophages, monocytes (Cell Bank of the Chinese Academy of Sciences, Shanghai, China), and neutrophils (Cell Bank of the Chinese Academy of Sciences) were seeded on HA and H/CS‐MOS hydrogels in 6‐well plates at a density of 5 × 10⁶ cells per well. After a 4‐day culture, EVs were collected as previously described and quantified using NTA to measure their relative abundance.

### Reparative Potential of EVs from Different Hydrogel‐Cultured Cells on RA Chondrocytes

EVs from M1‐induced BMMs, monocytes, and neutrophils, which were cultured on HA and H/CS‐MOS hydrogels for 48 h, were diluted in DMEM with 2% EV‐depleted serum to achieve a final concentration of 0.5 × 10⁹ mL⁻¹. The lyophilized hydrogels were immersed in this EV‐containing medium for 1 h to facilitate absorption. RA chondrocytes were then seeded onto these hydrogels at a density of 10⁵ cells mL^−1^. After 7 days of co‐culture, the RA chondrocytes were analyzed further. Protein expression was assessed using Western Blot analysis. The Western Blot procedure was similar to that previously described, with incubation using antibodies against Col II (ab34712, Abcam) and MMP13 (ab315267, Abcam).

### Reparative Potential of EVs from In Vivo Recruited Cells on RA Chondrocytes

EVs from hydrogel‐recruited cells were cultured on HA and H/CS‐MOS hydrogels using a method similar to the aforementioned procedure and co‐cultured with RA chondrocytes for seven days. a) Protein expression was assessed using Western Blot analysis, with incubation using antibodies against Col II, Sox 9 (ab185966, Abcam), ACAN (ab313636, Abcam), and MMP13. b) mRNA levels of Col II, Sox 9, ACAN, and MMP13 were analyzed using RT‐PCR. The RT‐PCR procedure was similar to that previously described. A list of primer sequences is appended in Table S2 (Supporting Information). c) Immunofluorescence staining was employed to observe the effects of EVs on the expression of COL II and MMP13 proteins in RA chondrocytes. Following fixing with 2.5% glutaraldehyde solution, the cells were treated with primary antibodies against COL II (ab34712, Abcam) and MMP13 (ab315267, Abcam) at 4 °C overnight. The cells were then treated with fluorescently labeled secondary antibodies (COL II: ab150077; MMP13: ab150075, abcam). Fluorescence images were subsequently observed using CLSM.

### Establishment of the RA CIA Rat Model

In this study, 66 male Wistar rats, each weighing ≈200 g, were used for in vivo experiments. 18 rats were for short‐term experiments and other 48 rats for long‐term experiments. All procedures involving animals were approved by the Shanghai Lab Animal Research Center (SYXK (Hu) 2020‐0005). An emulsion prepared by mixing bovine type II collagen in acetic acid with an equal volume of CFA was used to induce collagen‐induced arthritis (CIA) in the rats. A 0.1 mL dose of this emulsion was injected subcutaneously at the base of the tail, ≈2–3 cm from the tail's base. A booster injection of 0.1 mL of the same emulsion was administered one week later. Once the disease had developed, the rats were anesthetized with Zoleti 50 (Virbac, Carros, France) at a dosage of 1 mL kg^−1^ body weight. The surgical area was prepared by trimming the fur with scissors and disinfecting the skin with alcohol. A medial incision was made on the knee joint, followed by careful dissection of the skin and underlying fascia. Blunt dissection exposed the distal femur, and the joint capsule was incised to reveal the femoral condyle. The patella was dislocated laterally, and a full‐thickness osteochondral defect (1.5 mm deep, 2 mm in diameter) was created in the trochlear groove of the left femur, simulating advanced cartilage and bone damage typical of late‐stage rheumatoid arthritis. The ligament and patella were then repositioned, and the incision was sutured in layers. Subsequently, a polymer precursor solution and crosslinking agent were mixed and quickly injected into the articular cavity using a syringe. (Exception: In short‐term experiments, the control group consisted of crosslinked hyaluronic acid hydrogel, representing the non‐in situ gas‐releasing group, which was directly implanted after defect creation.) For the short‐term experiments, all rats were euthanized 3 h post‐surgery for analysis. For the long‐term experiments, the rats were housed under standard conditions post‐surgery and received intraperitoneal antibiotic injections for five consecutive days. Arthritis severity was assessed every three days, and joint diameter was measured every six days. At 28 days, half of the rats were euthanized for analysis, with the remaining rats euthanized at 56 days for further evaluation.

### Micro‐Computed Tomography (Micro‐CT), X‐Ray Imaging, and Magnetic Resonance Imaging (MRI)

4 and 8 weeks post‐surgery, femur samples (n = 6) were collected and fixed in 4% paraformaldehyde for 3 days at 4 °C. Micro‐CT scans were performed using a nanoVoxel‐2000 system (Sanying, Tianjin, China) for 3D rotational imaging of the femur samples. Following 3D reconstruction, parameters such as bone volume fraction (BV/TV), trabecular number (Tb.N), trabecular thickness (Tb.Th), and trabecular separation (Tb.Sp) were automatically quantified. From each group, X‐ray and MRI scans were conducted on samples using a Toshiba E7252 X‐ray device (Toshiba, Tokyo, Japan) and a CG NOVILA 7.0T MRI scanner (Chenguang, Shanghai, China) to monitor the formation of new tissue.

### Histological, Immunohistochemical (IHC), and Immunofluorescence (IF) Analyses

The samples were fixed in 4% paraformaldehyde for 3 days, then decalcified in 10% EDTA (Solarbio, Beijing, China) for 1 month. Subsequently, the samples were embedded in paraffin and cut into 7 µm sections. The morphology and organization of the newly formed tissue were observed using H&E, Safranin O‐Fast Green, and Toluidine blue staining. Immunohistochemistry (IHC) was performed to assess the deposition of type II collagen, Sox9, Acan, MMP13 (ab219620, Abcam), and iNOS (ab283655, Abcam). Combining the results from different types of pathological staining, the samples were scored according to the Osteoarthritis Research Society International (OARSI) standards (Table S4, Supporting Information). Immunofluorescence (IF) staining was employed to measure the expression levels of CD197 and CD206.

### ELISA Test

At the respective time points of the short‐term and long‐term experiments, blood serum was collected to evaluate inflammation. The cytokines IL‐1β, TNF‐α, TGF‐β1, and IL‐10 present in the serum were measured using ELISA kits, following the protocol outlined by the manufacturer. (For the short‐term experiment, IL‐1β and TNF‐α were analyzed, while for the long‐term experiment, IL‐1β, TNF‐α, IL‐10, and TGF‐β were assessed).

### Statistical Analysis

The results were presented as mean ± SD, with statistical significance defined as a p‐value < 0.05. All analyses were performed across a minimum of three independent experiments using Origin (8.0) software to ensure the robustness and validity of the findings. Sample size (n), p‐value, data normalization, and specific statistical tests for each experiment were clarified in the figure legends. The one‐way analysis of variance (ANOVA) analysis with Tukey's post‐test was employed to analyze differences among multiple groups, and a two‐tailed Student's t‐test was performed for the comparison between the two groups. All tests were two‐sided.

## Conflict of Interest

The authors declare no conflict of interest.

## Supporting information



Supporting Information

## Data Availability

The data that support the findings of this study are available from the corresponding author upon reasonable request.
