# Supplementary material for: Mannose Oligosaccharide‐Conjugated In Situ Pore‐Forming Injectable Hydrogels for Rheumatoid Arthritis Treatment by Reprogramming Macrophage Extracellular Vesicles
Source: Small Methods. 2025 Jul 15;10(3):2500605. doi: 10.1002/smtd.202500605 (PMC12893307; doi:10.1002/smtd.202500605)
Supplement: Supplementary file 1 — Supporting Information [file SMTD-10-2500605-s001.docx]

Supporting Information

**Mannose Oligosaccharide-conjugated *in situ* Pore-forming Injectable Hydrogels for Rheumatoid Arthritis Treatment by Reprogramming Macrophage Extracellular Vesicles**

*Anan Zhang, Yifan Ma, Yutong Liu, Shiyan Dong, Michelle Najarro Torres, Betty Y.S. Kim, Changsheng Liu, Lili Sun^*^, Yuan Yuan^*^, Wen Jiang^*^*

**Table S1.** The pore structure parameters of HA and H/CS-MOS hydrogels

| Hydrogel | Total porosity  (%) | Average pore size  (μm) |
| --- | --- | --- |
| HA | 84.93906 ± 2.81037 | 491 ± 25 |
| H-0.1CM | 85.61467 ± 3.86504 | 487 ± 36 |
| H-0.2CM | 85.93901 ± 4.18523 | 484 ± 54 |

**Table S2.** Primers for RT-PCR.

| Gene | Sequence (5’-3’) | |
| --- | --- | --- |
| *Glut-1* | Forward | CTTGCTTGTAGAGTGACGATC |
|  | Reverse | CAGTGATCCGAGCACTGCTC |
| *PISD* | Forward | CAACCTCAGCGAGTTCTTCC |
|  | Reverse | CCTGCTCCACCTCAGAGTTC |
| *TFAM* | Forward | CTGCCTTCCTCTAGCCCGGG |
|  | Reverse | GTAACAGCAGACAACTTGTG |
| *β-actin* | Forward | GGTGTGATGGTGGGAATGGG |
|  | Reverse | ACGGTTGGCCTTAGGGTTCAG |
| *GAPDH* | Forward | GAAGGTCGGTGTGAACGGAT |
|  | Reverse | CCCATTTGATGTTAGCGGGAT |
| *Col II* | Forward | CAGGATGCCCGAAAATTAGGG |
|  | Reverse | ACCACGATCACCTCTGGGT |
| *Sox 9* | Forward | AGTACCCGCATCTGCACAAC |
|  | Reverse | ACGAAGGGTCTCTTCTCGCT |
| *Acan* | Forward | GTGGAGCCGTGTTTCCAAG |
|  | Reverse | AGATGCTGTTGACTCGAACCT |
| *MMP13* | Forward | TGTTTGCAGAGCACTACTTGAA |
|  | Reverse | CAGTCACCTCTAAGCCAAAGAAA |

**Table S3.** Arthritis index (AI) scoring system

| Features | Score |
| --- | --- |
| No abnormalities, similar to normal rats | 0 |
| Mild redness and swelling in the hind ankle or forelimbs, or redness in a single toe | 1 |
| Moderate redness and swelling in the hind ankle or forelimbs, or redness in a single toe, or moderate swelling of the entire foot or toe. | 2 |
| Severe redness and swelling in both forelimbs and hind limbs, with the entire limb swollen but still able to bear weight. | 3 |
| Severe swelling in the forelimbs or hind limbs, with joint stiffness and inability to bear weight or touch the ground. | 4 |

**Table S4.** Osteoarthritis Research Society International (OARSI) score standards

| Grade | Osteoarthritic damage |
| --- | --- |
| 0 | Normal |
| 0.5 | Loss of Safranin-O without structural changes |
| 1 | Small fibrillations without loss of cartilage |
| 2 | Vertical clefts down to the layer immediately below the superficial layer and some loss of surface lamina |
| 3 | Verical clefts/erosion to the calcified cartilage extending to <25% of the articular surface |
| 4 | Vertical clefts/erosion to the calcified cartilage extending to 25–50% of the articular surface |
| 5 | Vertical clefts/erosion to the calcified cartilage extending to 50–75% of the articular surfac |
| 6 | Vertical clefts/erosion to the calcified cartilage extending >75% of the articular surface |


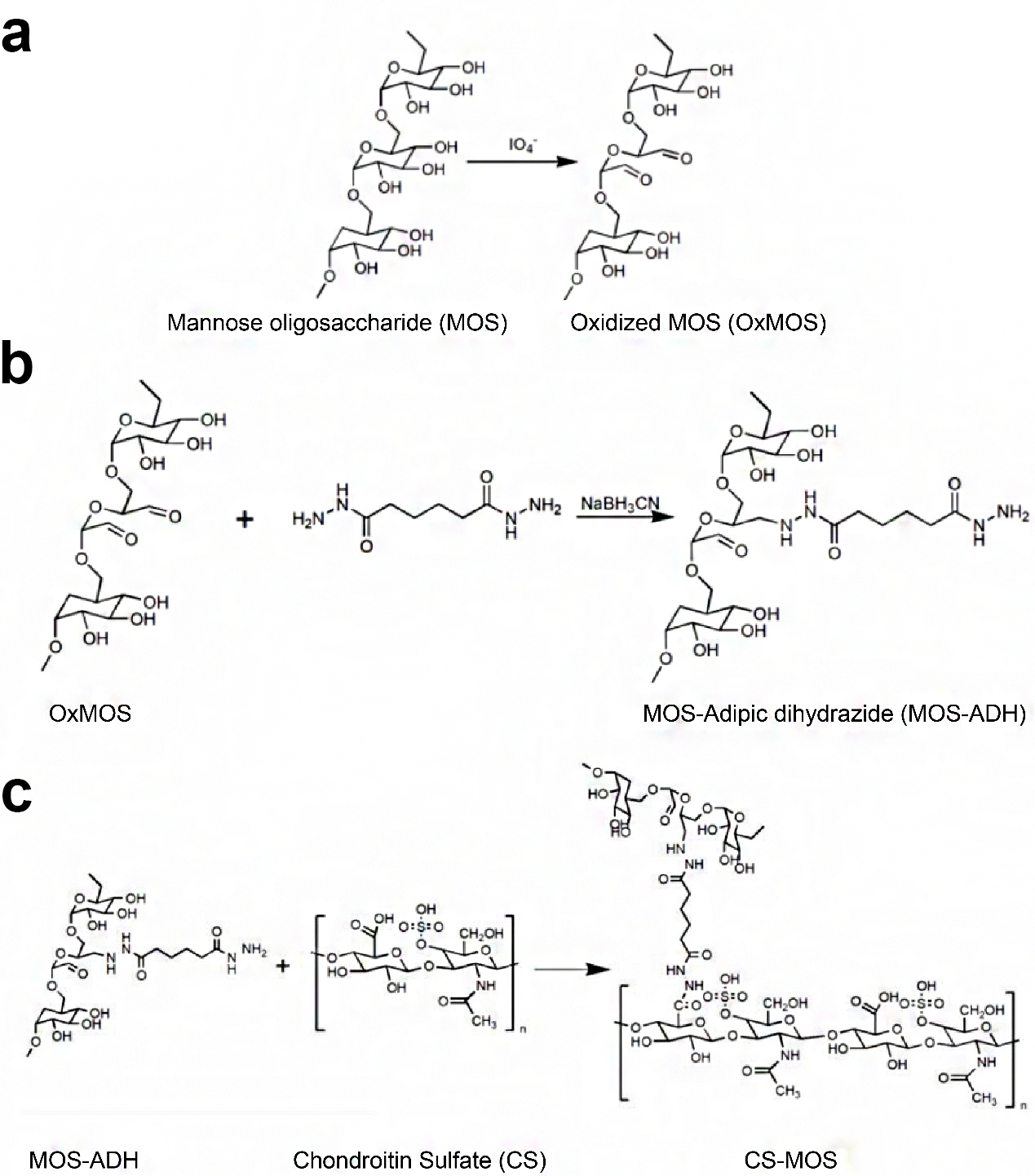


Figure S1. a) Synthetic scheme of OxMOS. b) Synthetic scheme of MOS-ADH. c) Synthetic scheme of CS-MOS.


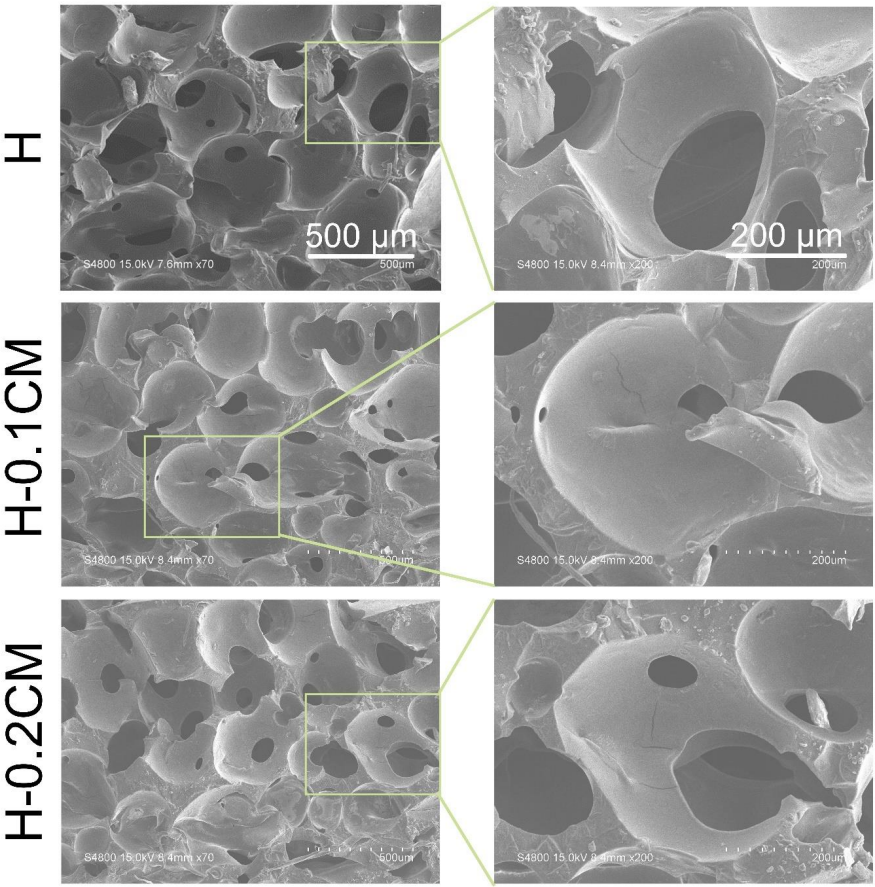


Figure S2. SEM images of lyophilized H/CS-MOS.


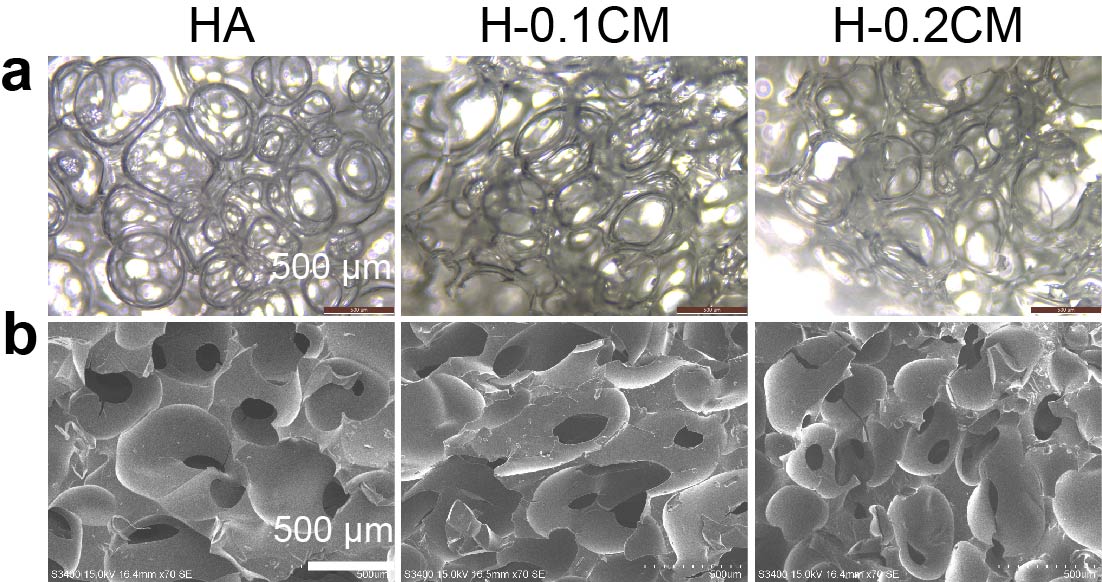


Figure S3. Micro-morphologies of bulk H/CS-MOS hydrogels. (a) Inverted microscope images and (b) Cryo-SEM images.

Figure S4. Compression strain of H/CS-MOS hydrogels.


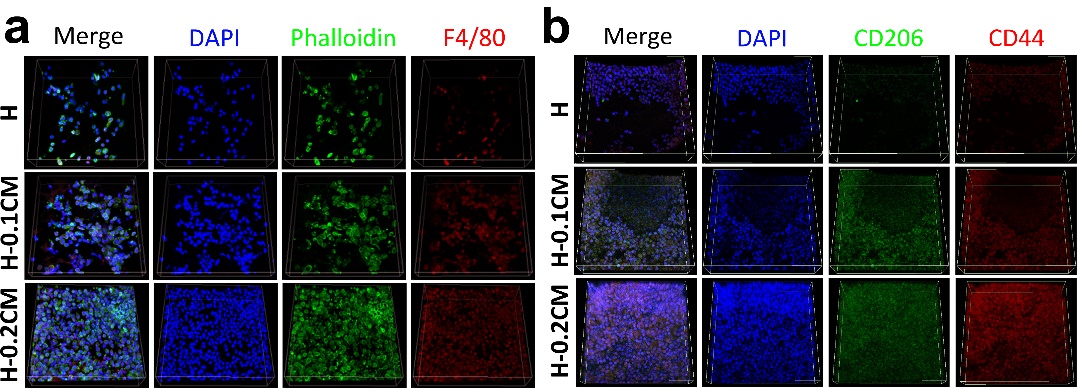


Figure S5. (a) CLSM images of F4/80-labeled macrophages recruited on the scaffolds. (b) CLSM images of CD44 and CD206-labeled M2 macrophages recruited on the scaffold.

Figure S6. Protein quantification of LC-3II compared to LC3-I. (n=3, ***p*<0.01)

Figure S7. Relative protein expression based on the Western blot results in Figure 4c. (CD9, CD63, and CD81 were normalized to the HA group, and Calnexin to the Ctrl group.) (n=3, ****p*<0.001, "n.s.": no significant difference)


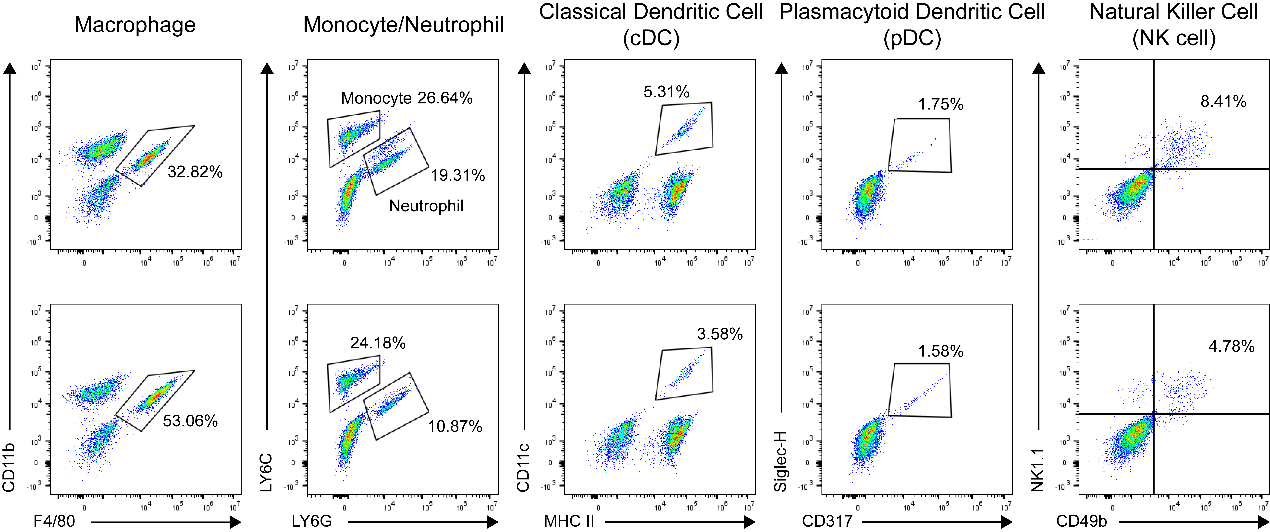


Figure S8. Flow cytometric analysis showing the proportion of the macrophages, monocytes, neutrophil, dendritic cells (cDC and pDC), and natural killer cell among the recruited cells.


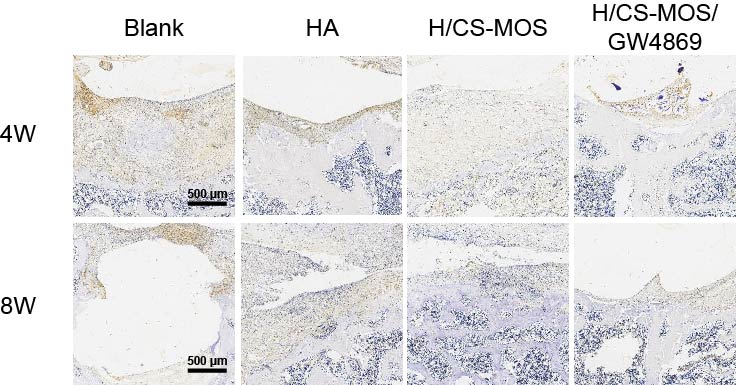


Figure S9. Immunohistochemical visualization of iNOS in the areas surrounding the defects.


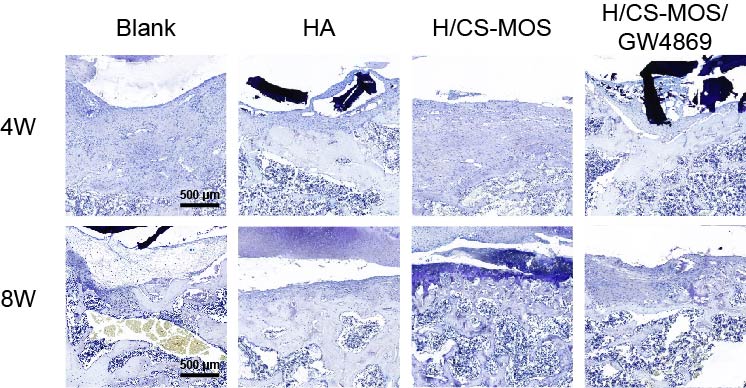


Figure S10. Toluidine blue staining of tissue sections surrounding the defects.
